# *De Novo* Synthesis of Phosphatidylcholine Is Essential for the Promastigote But Not Amastigote Stage in *Leishmania major*

**DOI:** 10.3389/fcimb.2021.647870

**Published:** 2021-03-12

**Authors:** Samrat Moitra, Somrita Basu, Mattie Pawlowic, Fong-fu Hsu, Kai Zhang

**Affiliations:** ^1^ Department of Biological Sciences, Texas Tech University, Lubbock, TX, United States; ^2^ Mass Spectrometry Resource, Division of Endocrinology, Diabetes, Metabolism, and Lipid research, Department of Internal Medicine, Washington University School of Medicine, St. Louis, MO, United States

**Keywords:** phospholipid, protozoan, salvage, amastigote, host

## Abstract

Phosphatidylcholine (PC) is the most abundant type of phospholipids in eukaryotes constituting ~30% of total lipids in *Leishmania*. PC synthesis mainly occurs *via* the choline branch of the Kennedy pathway (choline ⇒ choline-phosphate ⇒ CDP-choline ⇒ PC) and the N-methylation of phosphatidylethanolamine (PE). In addition, *Leishmania* parasites can acquire PC and other lipids from the host or culture medium. In this study, we assessed the function and essentiality of choline ethanolamine phosphotransferase (CEPT) in *Leishmania major* which is responsible for the final step of the *de novo* synthesis of PC and PE. Our data indicate that CEPT is localized in the endoplasmic reticulum and possesses the activity to generate PC from CDP-choline and diacylglycerol. Targeted deletion of *CEPT* is only possible in the presence of an episomal *CEPT* gene in the promastigote stage of *L. major*. These chromosomal null parasites require the episomal expression of *CEPT* to survive in culture, confirming its essentiality during the promastigote stage. In contrast, during *in vivo* infection of BALB/c mice, these chromosomal null parasites appeared to lose the episomal copy of *CEPT* while maintaining normal levels of virulence, replication and cellular PC. Therefore, while the *de novo* synthesis of PC/PE is indispensable for the proliferation of promastigotes, intracellular amastigotes appear to acquire most of their lipids through salvage and remodeling.

## Introduction

Leishmaniasis is caused by protozoan parasites of the genus *Leishmania* which alternate between extracellular promastigotes colonizing the midgut of sandflies and intracellular amastigotes residing in the macrophage of mammals (Alvar et al., 2012). Without a true vaccine, the mitigation of leishmaniasis mainly depends on vector control and drugs (Croft and Olliaro, 2011). Current therapeutics are limited and plagued with high toxicity (Croft and Olliaro, 2011). A better understanding of how *Leishmania* parasites acquire essential cellular components may lead to new drug targets and improved treatments.

Glycerophospholipids such as phosphatidylcholine (PC) and phosphatidylethanolamine (PE) are among the most abundant types of lipids accounting for approximately 30% and 10% of total lipids in *Leishmania*, respectively (Zhang and Beverley, 2010; Zheng et al., 2010). While PC in promastigotes primarily consists of 1,2-diacyl-PC, the majority of leishmanial PE belongs to 1-O-alk-1′-enyl-2-acyl-*sn*-glycero-3-phosphoethanolamine or plasmenylethanolamine (PME) (Beach et al., 1979; Zufferey et al., 2003; Pawlowic et al., 2016; Moitra et al., 2019). Due to its positively charged head group, PC is a membrane-forming phospholipid that is more abundant on the outer leaflet of the plasma membrane (van Meer et al., 2008; Nickels et al., 2015). Meanwhile, PE is known for its ability to promote membrane fusion and mainly resides in the inner leaflet (Verkleij et al., 1984; Ellens et al., 1989; Zachowski, 1993). Besides being principal membrane components, PC and PE possess other important functions. For example, PC can serve as the precursor for a number of important signaling molecules and metabolic intermediates including lyso-PC, phosphatidic acid, diacylglycerol (DAG), and free fatty acids (FA) (Exton, 1994; Furse and de Kroon, 2015). In addition, PE is required for the synthesis of GPI-anchored proteins in *Trypanosoma brucei* (a trypanosomatid parasite related to *Leishmania*) by providing the ethanolamine (EtN) phosphate bridge that links proteins to glycan anchors (Menon et al., 1993). PE also contributes to the formation of autophagosome during differentiation and starvation in *Leishmania major* (Besteiro et al., 2006; Williams et al., 2012) and the posttranslational modification of eukaryotic elongation factor 1A in *T. brucei* (Signorell et al., 2008a). Thus, understanding the mechanism by which *Leishmania* acquire their PC and PE may reveal new ways to block their growth.

**Figure 1:**
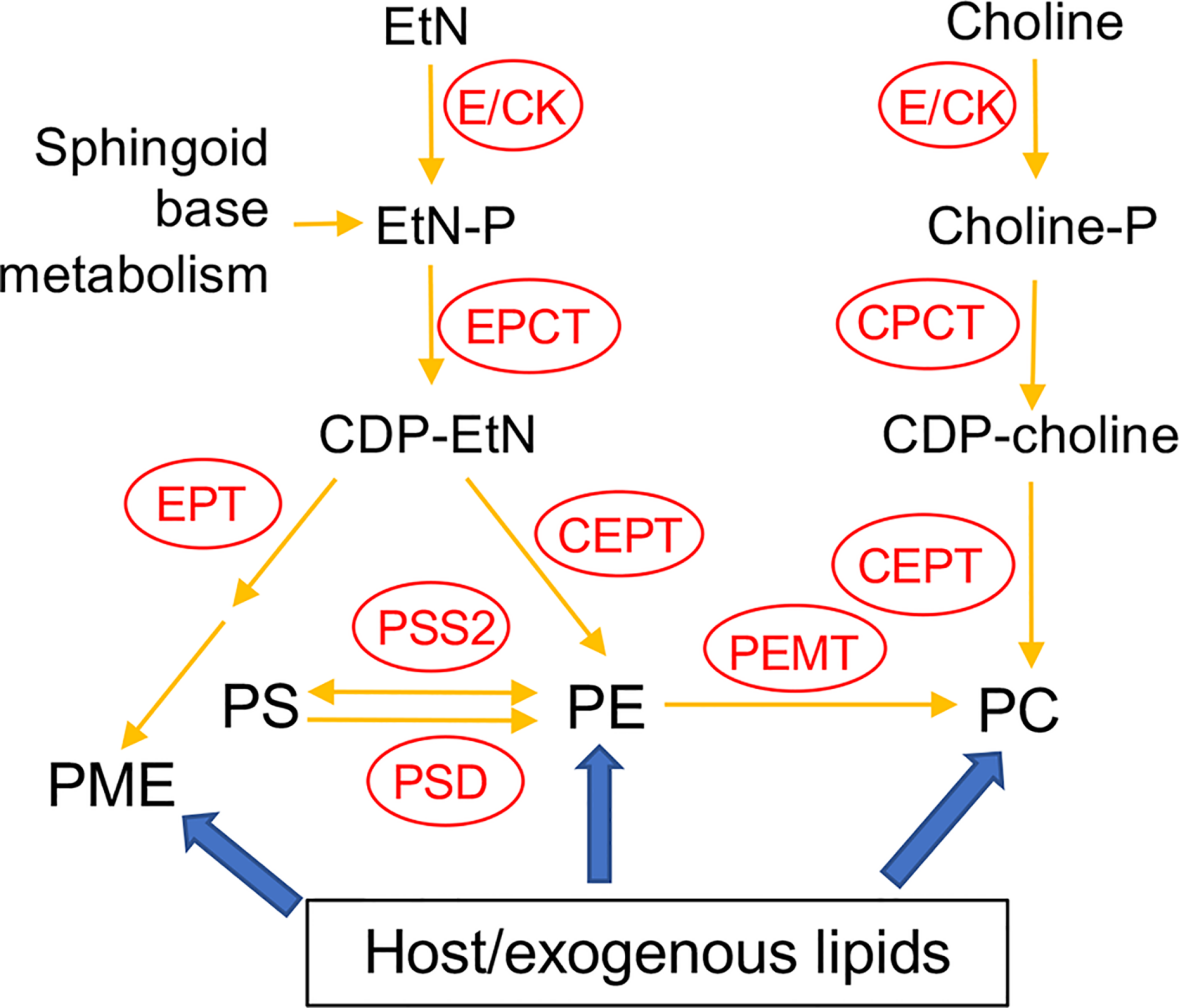
Acquisition of PE and PC in *Leishmania*. E/CK, ethanolamine/choline kinase; EPCT, ethanolaminephosphate cytidylyltransferase; EPT, ethanolamine phosphotransferase; CPCT, cholinephosphate cytidylyltransferase; CEPT, choline ethanolamine phosphotransferase; PEMT, phosphatidylethanolamine N-methyltransferase; PSS2, phosphatidylserine synthase 2; PSD, phosphatidylserine decarboxylase; EtN, ethanolamine; EtN-P, ethanolamine phosphate; PE, (1,2-diacyl-) phosphatidylethanolamine; PME, plasmenylethanolamine; Choline-P, choline phosphate; PC, phosphatidylcholine; PS, phosphatidylserine. Adapted from Moitra et al. (2019) and Pawlowic et al. (2016).

In many eukaryotic cells, the majority of PE and PC are synthesized *de novo via* the Kennedy pathway (Kennedy, 1956; Cui and Vance, 1996; Gibellini and Smith, 2010). As indicated in **Figure 1**, the *de novo* synthesis of PE, i.e. the EtN branch of the Kennedy pathway, starts with the phosphorylation of EtN into EtN-phosphate, which is also generated from the metabolism of sphingoid bases (Zhang et al., 2007); EtN-phosphate is then conjugated to CTP by ethanolaminephosphate cytidylyltransferase (EPCT) to produce CDP-EtN and pyrophosphate; and two enzymes catalyze the final steps: an ethanolamine phosphotransferase (EPT) which combines CDP-EtN with 1-alkyl-2-acyl-glycerol to form the precursor of PME (Pawlowic et al., 2016), and a choline ethanolamine phosphotransferase (CEPT) which condenses CDP-EtN and DAG into diacyl-PE. A parallel route, aka the choline branch of the Kennedy pathway, is responsible for the *de novo* synthesis of PC (choline → choline-phosphate → CDP-choline → PC), and CEPT catalyzes the last step of combining CDP-choline and DAG into PC as a dual activity enzyme (Henneberry and McMaster, 1999; Signorell et al., 2008b; Vance, 2008).

In addition to *de novo* synthesis, PE and PC are also generated from the conversion of phosphatidylserine (PS) and the N-methylation of PE, respectively (**Figure 1**). Furthermore, *Leishmania* can scavenge glycerophospholipids and degrade them *via* the activity of phospholipase A2 (PLA2), suggesting that these parasites can remodel exogenous lipids into their own *via* the Lands cycle (Das et al., 2001; Henriques et al., 2003; Parodi-Talice et al., 2003; Castanys-Munoz et al., 2007; Pawlowic and Zhang, 2012). In *T. brucei*, the Kennedy pathway is the dominant route for PE/PC synthesis (Signorell et al., 2008b; Gibellini and Smith, 2010). To explore the significance of this pathway in *L. major*, we first generated the *EPT*-null mutants (*ept¯*) which were largely devoid of PME but contained normal levels of diacyl-PE (Pawlowic et al., 2016). *Ept¯* promastigotes were fully viable and replicative in culture but showed significantly attenuated virulence in mice (Pawlowic et al., 2016). Meanwhile, *ept¯* amastigotes were fully virulent, indicating that PME synthesis alone was not required during the mammalian stage for *L. major* (Pawlowic et al., 2016). Second, to determine the role of *de novo* PC synthesis in *L. major*, we focused on the cholinephosphate cytidylyltransferase (CPCT) which catalyzes the choline-phosphate to CDP-choline conversion in the choline branch of the Kennedy pathway (**Figure 1**). Without CPCT, *L. major* parasites could not incorporate choline into PC, yet the *CPCT*-null promastigotes (*cpct¯*) contained similar levels of PC and PE as wild type (WT) parasites in culture (Moitra et al., 2019). Loss of *CPCT* did not affect promastigote replication in complete media but caused reduced growth rates under EtN-limiting conditions (Moitra et al., 2019). Both *cpct¯* promastigotes and amastigotes were fully virulent in mice (Moitra et al., 2019). Collectively, these observations suggest that other routes of PC synthesis (e.g., PE N-methylation and lipid salvage) can compensate the loss of CPCT (**Figure 1**).

In this study, we investigated the function of CEPT in *L. major* which is expected to directly catalyze the formation of diacyl-PC and diacyl-PE *via* the Kennedy pathway (**Figure 1**). In *T. brucei*, RNAi knockdown of CEPT significantly reduced the biosynthesis of PE and PC and caused a cytokinesis defect resulting in the accumulation of zoids (Signorell et al., 2008b; Signorell et al., 2009). These findings are in agreement with the dual substrate activity exhibited by *T. brucei* CEPT (Farine et al., 2015). Given the fact that CPCT is not essential for *L. major*, it is important to probe whether the *de novo* PC synthesis is required for *Leishmania* promastigotes and amastigotes. Unlike *T. brucei* which has separate Kennedy pathway branches for PE and PC synthesis (Gibellini and Smith, 2010; Smith and Butikofer, 2010), *Leishmania* parasites can incorporate EtN into both PE and PC (Pawlowic et al., 2016; Moitra et al., 2019), presumably through the activity of PE-N-methyltransferases (Bibis et al., 2014). Thus, CEPT is likely required for the synthesis of PC through both the Kennedy pathway and the methylation of PE in *Leishmania* (**Figure 1**). Our results demonstrate that CEPT is indispensable for *L. major* promastigotes but not amastigotes, suggesting that intracellular parasites can fulfill their need for PC through lipid salvage/remodeling.

## Materials and Methods

### Materials

For the CEPT activity assay, [methyl-^14^C] CDP-choline (50–60 mCi/mmol) and [diarachidonyl-1-^14^C] PC (50–60 mCi/mmol) were purchased from Perkin Elmer, Inc (Waltham, MA) and American Radiolabeled Chemicals (St. Louis, MO), respectively. 1,2-Dioctanoyl-sn-glycerol (DAG) was purchased from EMD Millipore (Burlington, MA). Lipid standards for mass spectrometry including 1,2-dimyristoyl-*sn*-glycero-3-phosphoethanolamine (14:0/14:0-PE) and 1,2-dimyristoyl-*sn*-glycerol-3-phosphocholine (14:0/14:0-PC) were purchased from Avanti Polar Lipids (Alabaster, AL). All other reagents were purchased from Thermo Fisher Scientific (Hampton, NH) unless specified otherwise.

### Molecular Constructs

The open reading frame (ORF) of *CEPT* (LmjF36.5900) was amplified by PCR from *L. major* genomic DNA and cloned into the pXG vector (Ha et al., 1996) to generate pXG*-CEPT*. The *CEPT* ORF was then subcloned into the pXG-GFP’ (Ha et al., 1996) and pXNG4 vectors (Murta et al., 2009) to generate pXG-*GFP-CEPT* and pXNG4*-CEPT*, respectively. The upstream and downstream flanking sequences (~1 Kb each) of *CEPT* were PCR amplified and cloned in tandem into the pUC18 vector. Genes conferring resistance to puromycin (*PAC*) and blasticidin (*BSD*) were inserted between the upstream and downstream flanking sequences to generate pUC18-KO*-CEPT PAC* and pUC18-KO*-CEPT BSD*, respectively. All the molecular constructs were confirmed by restriction enzyme digestion and sequencing. Oligo nucleotides used in this study are summarized in **Table S1**.

### *Leishmania* Culture and Genetic Manipulation

*L. major* LV39 clone 5 (Rho/Su/59/P) promastigotes were cultivated at 27°C in M199 media with 10% heat-inactivated fetal bovine serum and other supplements as previously described (Kapler et al., 1990a). To monitor cell growth, promastigotes were inoculated at 1.0–2.0 × 10^5^ cells/ml and culture densities were determined over time using a hemocytometer. In general, log phase promastigotes refer to replicative parasites at densities lower than 1.0 × 10^7^ cells/ml and stationary phase promastigotes refer to mainly non-replicative parasites at 2.0–3.0 × 10^7^ cells/ml. The proliferation of promastigotes from 1.0–2.0 × 10^5^ cells/ml to 2.0–3.0 × 10^7^ cells/ml (usually in 2–3 days) is referred to as one passage.

To delete *CEPT*, antibiotic resistance cassettes acquired from pUC18-KO*-CEPT PAC* and pUC18-KO*-CEPT BSD* were sequentially introduced into LV39 wild type (WT) parasites and drug selections were performed as previously described (Kapler et al., 1990a). With this approach, we were able to generate the heterozygous *CEPT+/−* mutants (*ΔCEPT::PAC/CEPT*) but not the homozygous *cept —* mutants (*ΔCEPT::PAC/ΔCEPT::BSD*). To delete the second chromosomal allele of *CEPT*, the pXNG4*-CEPT* plasmid containing *GFP*, *TK*, *SAT* (nourseothricin resistance), and *CEPT* genes was introduced into *CEPT+/−* parasites. The resulting *CEPT+/−* +pXNG4*-CEPT* cells were transfected with the blasticidin resistance cassette from pUC18*-CEPT-KO BSD*. Parasite showing resistance to puromycin, blasticidin, and nourseothricin were serially diluted into 96-well plates. The resulting clones were amplified and processed for Southern blot analysis to verify the loss of chromosomal *CEPT* and referred to as *cept —* +pXNG4*-CEPT*.

### Fluorescence Microscopy and Validation of GFP*-CEPT*

Promastigotes expressing GFP*-CEPT* were attached to poly-L-lysine coated coverslips, fixed with 3.7% formaldehyde, and then permeabilized on ice with ethanol. Incubation with rabbit anti-*T. brucei* BiP antiserum (1:1,000) was performed at room temperature for 40 min. After washing three times with phosphate-buffered saline (PBS), coverslips were incubated with a goat anti-rabbit-IgG Texas Red (1:2,000) antiserum for 40 min. After washing three times with PBS, DNA was stained with 2.5 µg/ml of Hoechst 33342, followed by final three washes with PBS. Coverslips containing cells were mounted on glass slides with Fluormount-G mounting medium and an Olympus BX51 Upright Fluorescence Microscope was used to visualize the expression and localization of GFP*-CEPT*. To quantify the overlap between GFP*-CEPT* and anti-BiP staining, 136 randomly selected cells were analyzed using the Image J JACoP (Just Another Colocalization Plugin) (**Table S2**) (Bolte and Cordelieres, 2006).

To detect GFP*-CEPT* by Western blot, *Leishmania* promastigotes were boiled in SDS sample buffer, resolved by SDS-PAGE and probed with a rabbit anti-GFP polyclonal antibody (Life Technologies) followed by a goat anti-rabbit IgG-HRP antibody. To ensure equal loading, blots were probes with an anti-α-tubulin monoclonal antibody followed by a goat anti-mouse IgG-HRP antibody. Signals from Western blot were detected using a FluorChem E system (Protein Simple).

### CEPT Assay and Thin Layer Chromatography (TLC)

To examine the CEPT activity in *Leishmania*, log phase promastigotes of WT, *CEPT+/− +*pXG*-CEPT*, and WT *+*pXG-*GFP-CEPT* were harvested by centrifugation, washed twice with PBS, and resuspended in a hypotonic lysis buffer (1 mM of Tris-HCl, 0.1 mM of EDTA, 1 × protease inhibitor cocktail, pH 8) at 5 × 10^8^ cells/ml. Cells were lysed by sonication (Ultrasonic homogenizer Sonic Ruptor 250, medium speed, 30 s × three times on ice) and protein concentration in lysate was determined by the BCA assay. Procedure for the CEPT activity assay was adapted from a previously reported method with *Trypanosoma brucei* cells (Farine et al., 2015). Briefly, each 100-μl reaction contained 900 μg of leishmanial protein (from promastigote lysate), 50 μM of [^14^C] CDP-choline, 50 μM of DAG, 50 mM of Tris pH 8.0, 10 mM of MgCl_2_, and 0.005% of Tween 20 (w/v). After 1-h incubation at room temperature, the reaction mix was extracted twice with of 250 μl of 1-butanol. The combined organic fractions were dried under a nitrogen stream and reconstituted in 20 μl of 1-butanol. The aqueous fraction was also saved. Materials from the organic and aqueous fractions were analyzed by one-dimensional TLC on a silica gel 60 plate (Merck) using a solvent system composed of chloroform:methanol:acetate:water (25:15:4:2 by volumes). [^14^C] CDP-choline, [^14^C] 1,2-diarachidonyl PC, and [^14^C] choline chloride were used in the TLC as markers. Mouse liver homogenate was used as a positive control and boiled *Leishmania* lysates were used as negative controls. After TLC, radioactive signals on the silica gel 60 plate were detected using a Personal Molecular Imager (Bio-Rad).

### Ganciclovir (GCV) Treatment of Promastigotes in Culture

Promastigotes of WT, *CEPT+/−* +pXNG4*-CEPT*, and *cept —* +pXNG4*-CEPT* were cultivated in complete M199 media in the absence or presence of 50 μg/ml of GCV or 200 μg/ml of nourseothricin (for *CEPT+/−* +pXNG4*-CEPT* and *cept —* +pXNG4*-CEPT* only). Every three days, parasites were re-inoculated at 1.0 × 10^5^ cells/ml in fresh media containing the same concentration of GCV or nourseothricin to start a new passage. For every passage, the GFP expression level was determined by flow cytometry at mid-log phase (3–7 × 10^6^ cells/ml) using an Attune NxT Acoustic Flow Cytometer. After 16 passages in the presence of 50 μg/ml of GCV, *cept — +*pXNG4*-CEPT* parasites were sorted based on their GFP expression levels using a BD Biosciences FACS Aria III Plus Cell Sorter. GFP-high and GFP-low parasites (~50,000 each) were sorted directly into complete M199. Parasites showing low GFP expression were serially diluted into 96-well plates. Multiple clones were then selected and their GFP expression levels were determined by flow cytometry (examples are shown in **Figures 5E–G**).

### Quantitative PCR (qPCR) Analyses to Determine *CEPT* Transcript Level, Parasite Loads, and Plasmid Copy Numbers

To determine the *CEPT* transcript level in WT parasites, total RNA was extracted from log phase promastigotes or lesion-derived amastigotes. One microgram of RNA was converted into complementary DNA using a high-capacity cDNA conversion kit, followed by qPCR using primers designed for the ORF of *CEPT* or the 28S rRNA gene. The expression level of *CEPT* was normalized to that of 28S rRNA using the comparative Ct approach, also known as the 2^−ΔΔ (Ct)^ method (Livak and Schmittgen, 2001).

To determine parasite numbers, a standard curve was prepared from the serially diluted genomic DNA samples extracted from WT promastigotes (from 2 × 10^6^ cells to 0.2 cells per reaction). Dilutions were carried out in the presence of salmon sperm DNA as a carrier. QPCR was performed on DNA extracted from promastigotes and amastigotes with primers for the 28S rRNA gene. Parasite numbers were determined using the standard curve.

To determine pXNG4*-CEPT* copy numbers, a calibration curve was generated by serially diluting pXNG4*-CEPT* plasmid DNA from 2 × 10^6^ molecules to 0.2 molecules per reaction in the presence of salmon sperm DNA. QPCR was performed on DNA extracted from promastigotes and amastigotes using primers for the pXNG4 plasmid. Plasmid copy number per cell was calculated by dividing the total plasmid copy number by the total parasite number. All qPCR reactions were performed in triplicates using the SSO Advanced Universal SYBR Green Supermix (Bio-Rad) in an Applied Biosystems 7300 RT-PCR thermo-cycler.

### Mouse Footpad Infection and *In Vivo* GCV Treatment

Use of mice in this study was approved by the Animal Care and Use Committee at Texas Tech University. BALB/c mice (female, 8 weeks old) were purchased from Charles River Laboratories International. To assess parasite virulence, stationary phase promastigotes were resuspended in serum free M199 medium and injected into the left hind footpad of BALB/c mice (1.0 × 10^6^ cells/mouse, 10 mice per group). For each group, one half of the mice received GCV treatment at 7.5 mg/kg/day (GCV was prepared in sterile PBS, 0.5 ml per day) through intraperitoneal injection, while the other half received sterile PBS (0.5 ml per day). GCV and PBS treatments started one day post infection and continued for 14 days. Mouse body weights were monitored once a week for three weeks post injection. Footpad lesions were measured weekly using a Vernier caliper. Parasite loads in infected footpads were determined at the indicated times by limiting dilution assay as described previously (Titus et al., 1985) and qPCR as described above. Mice were euthanized *via* CO_2_ asphyxiation when their footpad lesions exceeded 2.5 mm or when secondary infections were detected.

### Recovery and Analyses of Promastigotes From Infected Mice

Amastigotes were isolated from 8–10 weeks infected mice and allowed to convert into promastigotes in complete M199 media in the presence or absence of 200 μg/ml of nourseothricin or 50 μg/ml of GCV. Parasites were then continuously cultivated in media containing the same concentration of nourseothricin or GCV. During each passage, the growth rate and GFP expression were determined by cell counting and flow cytometry as described above.

### Lipid Analysis by Electrospray Ionization Mass Spectrometry (ESI-MS)

Total lipids from log phase promastigotes, lesion-derived amastigotes, or uninfected mouse tissue were extracted using the Bligh-Dyer method (Bligh and Dyer, 1959; Zhang et al., 2012). Purification of lesion amastigotes was performed as previously described (Glaser et al., 1990; Zhang et al., 2012). Identification of PC, PE and TAG structures using low-energy collision induced dissociation LIT MS^n^ with high-resolution Fourier transform mass spectrometry was conducted on a Thermo Scientific LTQ Orbitrap Velos mass spectrometer (R=100,000 at *m/z* 400) with Xcalibur operating system as previously described (Hsu et al., 2014). Fatty acyl constituents and numbers of double bonds in PC, PE and TAG were given when possible. For example, *a*16:0/16:0 PC represents 1-O-hexadecyl-2-palmitoyl-sn-glycero-3- phosphocholine, *e*18:0/18:2-PE represents 1-O-octadec-1′-enyl-2-octadecadienoyl-sn-glycero-3-phosphoethanolamine, and 36:4 PC represents a 1,2-diacyl-sn-glycero-3-phosphocholine in which the total number of carbon from sn-1 and sn-2 is 36 and the total number of C=C double bonds from sn-1 and sn-2 is 4, and 52:3 TAG represents a TAG in which the total number of carbon from sn-1 to sn-3 is 52 and the total number of C=C double bonds is 3.

### Statistical Analysis

Unless otherwise specified, all experiments were repeated three times and each biological repeat contained 2–3 technical repeats. Differences among experimental groups were determined by the unpaired Student’s *t* test (for two groups) or one-way ANOVA (for three to four groups) using Sigmaplot 13.0 (Systat Software Inc, San Jose, CA). *P* values indicating statistical significance were grouped into values of <0.05 (*), <0.01 (**) and <0.001 (***).

## Results

### Targeted Deletion of the Endogenous *CEPT* Alleles in *L. major*

**Figure 2:**
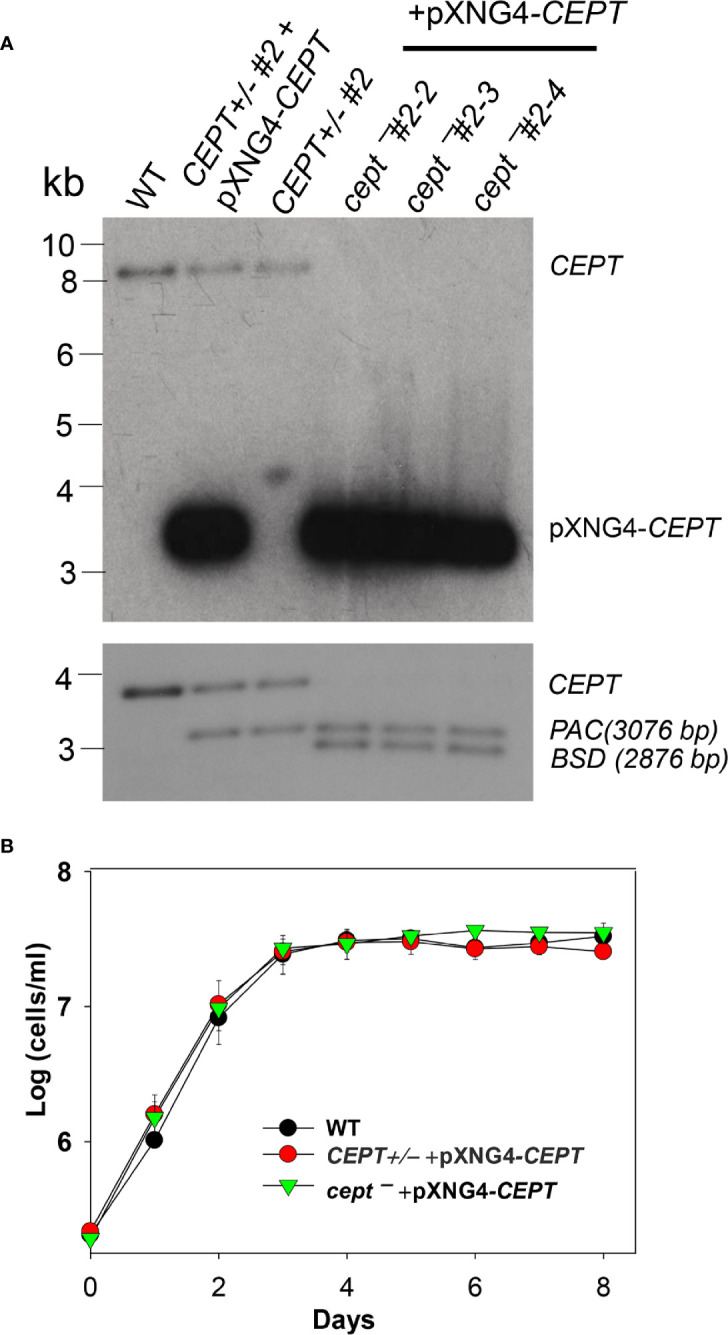
Deletion of *L. major CEPT* in the presence of pXNG4-*CEPT*. **(A)** Genomic DNA samples from WT, *CEPT +/−* clone #2, *CEPT +/−* +pXNG4-*CEPT* (clone #2), and *cept¯ +*pXNG4-*CEPT* (clones #2-3, 2-3, and 2-4) parasites were processed for Southern blot using radiolabeled probes for the ORF (top) or flanking sequence (bottom) of *CEPT*. Bands corresponding to chromosomal *CEPT* (top: 8252 bp, bottom: 3730 bp), pXNG4-*CEPT* (top: 3475 bp), *PAC* (bottom: 3076 bp) and *BSD* (bottom: 2876 bp) were indicated. **(B)** Promastigotes of WT, *CEPT+/−* +pXNG4-*CEPT*, and *cept¯ +*pXNG4-*CEPT* were cultivated at 27°C in complete M199 media and culture densities were determined using a hemocytometer. Error bars represent standard deviations from three experiments.

*L. major* CEPT is encoded by a single copy gene on chromosome 36 (TritrypDB ID: LmjF36.5900) with well conserved, syntenic orthologs among other trypanosomatids. The predicted protein contains 417 amino acids with six transmembrane helices showing 30–33% identity to the CEPTs from organisms outside of kinetoplastids. To investigate the function of this enzyme in *L. major*, we first tried to generate the *CEPT*-null mutants using a classic approach based on homologous recombination (Cruz et al., 1991). With this method, we were able to replace the first *CEPT* allele with the puromycin resistance gene (*PAC*) generating *CEPT+/−* parasites (**Figure 2A**). However, repeated attempts to delete the second *CEPT* allele with the blasticidin resistance gene (*BSD*) were unsuccessful, suggesting that CEPT was indispensable for promastigotes. To overcome this obstacle, we used an episome facilitated approach to acquire the chromosomal knockouts (Murta et al., 2009). Briefly, *CEPT* was cloned into the pXNG4 vector which contains genes for nourseothricin resistance (*SAT*), green fluorescent protein (GFP), and thymidine kinase (TK). The pXNG4*-CEPT* plasmid was then introduced into *CEPT+/−* to generate *CEPT+/− +*pXNG4*-CEPT*, followed by the replacement of the second chromosomal *CEPT* allele with *BSD*. With this method, parasites showing resistance to puromycin, nourseothricin, and blasticidin were readily obtained. Individual clones were isolated from these parasites by serial dilution. In a Southern blot analysis, these clones (*cept¯ +*pXNG4*-CEPT* #2-2, 2-3, and 2-4) showed a complete loss of chromosomal *CEPT* while maintaining a high level of episomal *CEPT* (**Figure 2A**). No significant difference was detected among these clones and results from *cept¯ +*pXNG4*-CEPT* #2-3 were described in the following experiments. As shown in **Figure 2B**, overexpression of *CEPT* had little impact on promastigote replication in culture. The fact that chromosomal null mutants could only be generated in the presence of a complementing plasmid suggests that CEPT is essential during the promastigote stage of *L. major.*

### Expression and Localization of CEPT in *L. major*

**Figure 3:**
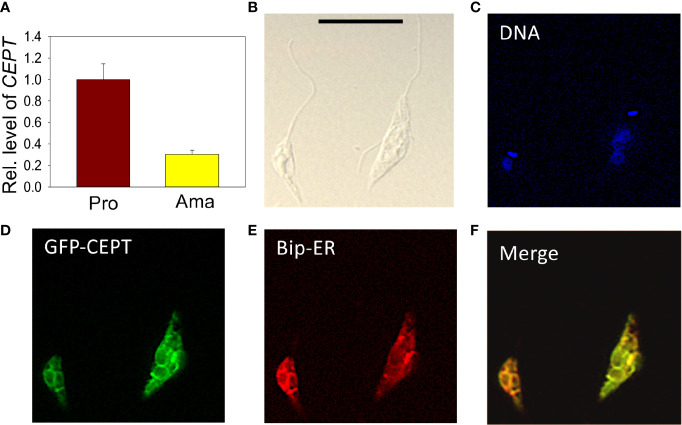
Transcript level and localization of CEPT in *L. major*. **(A)** The transcript level of *CEPT* was determined from culture promastigotes and lesion-derived amastigotes by qRT-PCR. The relative abundance of *CEPT* transcript was normalized using 28S RNA as the internal control based on ΔΔCt values. Error bars represent standard deviations from two independent biological repeats. **(B–F)** Log phase promastigotes of WT*+*pXG*-GFP-CEPT* were labeled with rabbit *anti-T. brucei* BiP antiserum followed by a goat anti-rabbit IgG-Texas Red antibody and subjected to immunofluorescence microscopy. **(B)** Differential interference contrast; **(C)** DNA staining; **(D)** GFP fluorescence; **(E)** anti-BiP staining; **(F)** merge of **(D, E)** Scale bar: 10 µm. The overlap between BiP and GFP-CEPT was determined by the JaCOP ImageJ analysis of 136 cells (**Table S1**).

The relative transcript levels of *CEPT* in wild type (WT) promastigotes (from *in vitro* culture) and amastigotes (isolated from infected BALB/c mice) were examined by qRT-PCR. Using the 28S RNA as the internal standard, we detected a three-fold reduction in *CEPT* transcript level in amastigotes in comparison to promastigotes (**Figure 3A**), suggesting that this enzyme is in lower demand during the intracellular stage. In *T. brucei*, EPT and CEPT are located in different (although overlapping) sub-compartments of the endoplasmic reticulum (ER), suggesting a spatial segregation of ether lipids (e.g. PME) and diacyl-PE/PC synthesis (Farine et al., 2015). To determine the cellular localization of CEPT in *L. major*, GFP*-CEPT* was introduced into WT promastigotes (**Figure S1**). In immunofluorescence microscopy, GFP*-CEPT* exhibited a diffused and membranous pattern resembling that of the ER marker BiP (**Figures 3B–F**). Quantitative analysis revealed ~73% overlap between GFP*-CEPT* and BiP (**Table S2**), suggesting that CEPT is primarily located at the bulk ER. This is similar to the localization of EPT and CPCT in *L. major* (Pawlowic et al., 2016; Moitra et al., 2019).

### CEPT Is Responsible for PC Synthesis From CDP-Choline and DAG

**Figure 4:**
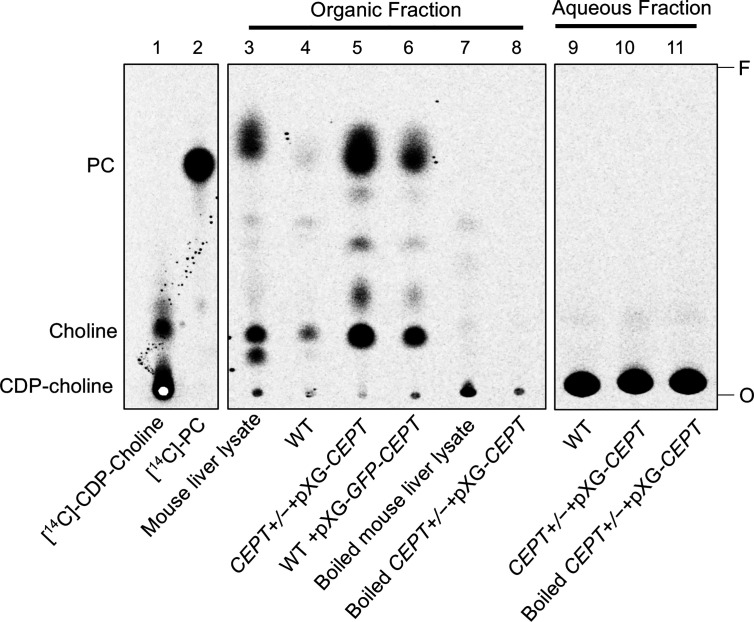
CEPT is responsible for the synthesis of PC from CDP-choline and DAG. Mouse liver lysate or promastigote lysate were incubated with [^14^C]-CDP-choline and DAG followed by extraction with 1-butanol and TLC analysis as described in *Materials and Methods*. Lane 1: [^14^C]-CDP-choline. Lane 2: [^14^C]-1,2-diarachidonyl PC. Lanes 3–8: Organic fractions after 1-butanol extraction. Lanes 9–11: aqueous fractions after 1-butanol extraction. O: origin. F: Solvent front. This assay was repeated three times and results from one representative experiment were shown here.

To determine whether *L. major* CEPT catalyzes the synthesis of PC, promastigote lysates were incubated with [^14^C]-CDP-choline and DAG for 1 h followed by lipid extraction and thin layer chromatography (TLC). As illustrated in **Figure 4**, overexpression of *CEPT* or *GFP-CEPT* led to robust production of [^14^C]-PC in the organic fraction. CEPT activity was also present in the mouse liver lysate (positive control). In comparison, this activity was barely detectable in WT parasites and completely absent in heat-inactivated lysates. Hydrolysis of CDP-choline into choline was also detected from unboiled samples. As expected, the majority of [^14^C]-CDP-choline (a substrate) was found in the aqueous phase (**Figure 4**). These findings suggest that CEPT is a functional enzyme capable of generating PC from CDP-choline and DAG in *L. major.* We were unable to confirm the contribution of CEPT to PE synthesis due to the lack of commercially available, radiolabeled CDP-EtN.

### CEPT Is Indispensable for *L. major* Promastigotes

**Figure 5:**
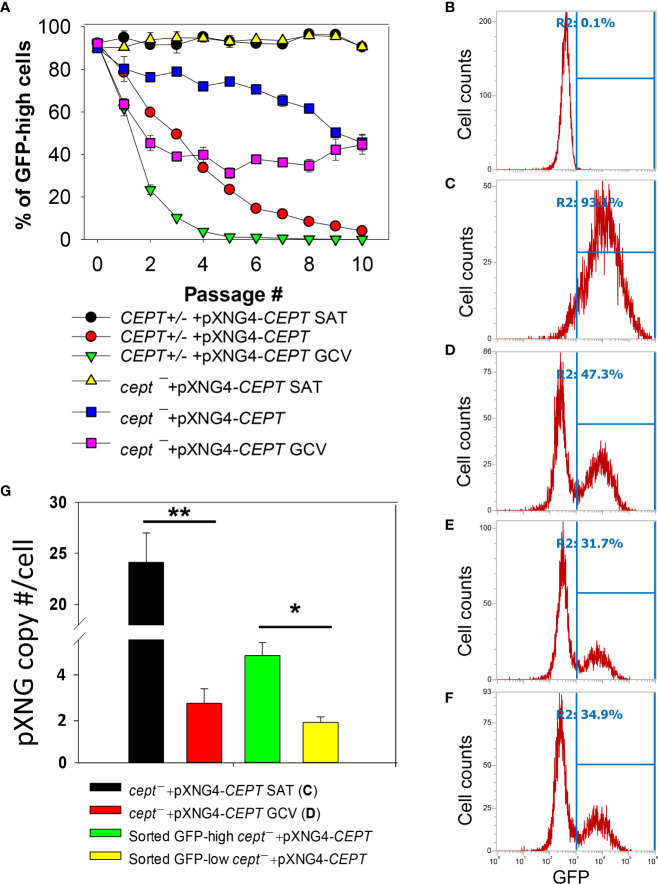
CEPT is indispensable for *L. major* promastigotes.** (A)** Promastigotes were continuously cultivated in the presence or absence of GCV or nourseothricin (SAT) and percentages of GFP-high cells were determined by flow cytometry for each passage. **(B–D)** After 16 passages, flow cytometry analyses were performed on WT parasites **(B)**, *cept¯*+pXNG4-*CEPT* parasites grown in the presence of nourseothricin **(C)** or GCV **(D)**. **(E, F)** Two clones of *cept¯*+pXNG4-*CEPT* were isolated from the GFP-low population in **(D)** by serial dilution and subjected to flow cytometry. In **(B–F)**, percentages of GFP-high cells (summarized in **A**) were indicated as R2. **(G)** The GFP-high and GFP–low parasites in **(D)** were separated by FACS, and plasmid copy numbers were determined by qPCR as described in *Materials and Method*s. Error bars represent standard deviations from two or three biological repeats (*p<0.05, **p<0.01).

To critically evaluate whether CEPT is required for *L. major* promastigotes, ganciclovir (GCV) was added to *CEPT+/−* +pXNG4*-CEPT* and *cept¯* +pXNG4*-CEPT* parasites which would trigger the formation of GCV-triphosphate through the activity of TK, resulting in premature termination of DNA synthesis. If the episomal copy of CEPT in pXNG4*-CEPT* is dispensable, it would be gradually lost during *in vitro* culture in the presence of GCV (Murta et al., 2009). This process was monitored by flow cytometry (the pXNG4 plasmid contains GFP) and qPCR over multiple passages in culture (**Figure 5**). As expected, when cultivated in the presence of nourseothricin (SAT) and absence of GCV, 90–95% of *CEPT+/−* +pXNG4*-CEPT* and *cept¯* +pXNG4*-CEPT* promastigotes showed high GFP expression by flow cytometry (**Figure 5A**). When cultivated in the absence of GCV or nourseothricin, the *CEPT+/−+*pXNG4*-CEPT* parasites gradually lost the episome from 92% GFP-high in passage 1 to <4% in passage 10 (**Figure 5A**). In the presence of GCV and absence of nourseothricin, the reduction of GFP fluorescence was much faster in these parasites (<1% GFP-high by passage 5, **Figure 5A**). Meanwhile, the *cept¯+*pXNG4*-CEPT* parasites were 40–50% GFP-high after 10 passages in the absence of GCV (**Figure 5A**). Importantly, continuous GCV treatment did not significantly reduce the GFP-high percentage in these cells (**Figures 5A–D**). Thus, unlike the *CEPT+/−+*pXNG4*-CEPT* parasites which had a chromosomal *CEPT* allele, *cept¯ +*pXNG4*-CEPT* parasites could not fully lose the episome even when they were under strong negative selection pressure.

Next, we examined if it was possible to isolate viable and replicative *CEPT*-null promastigotes without the episome. First, *cept¯+*pXNG4*-CEPT* parasites were continuously cultivated in the presence of GCV and absence of nourseothricin for 16 passages. As shown in **Figure 5D**, ~53% of these cells were classified as GFP-low and ~47% were classified as GFP-high. We then separated the GFP-low and GFP-high populations by fluorescence-activated cell sorting (FACS), followed by serial dilution to isolate clones from these populations. Importantly, when these GFP-low clones were cultivated in drug-free M199 media, 31–35% of them were GFP-high (**Figures 5E, F**). Furthermore, qPCR analyses revealed averages of 1.64 copies of pXNG4*-CEPT* plasmid per cell in the sorted GFP-low clones and 4.36 copies per cell in the sorted GFP-high clones (**Figure 5G**). Meanwhile, the pre-sorting population in **Figure 5D** (*cept¯+*pXNG4*-CEPT* grown in GCV for 16 passages) had 2.42 copies/cell (**Figure 5G**). As a control, *cept¯+*pXNG4*-CEPT* promastigotes cultured in the presence of nourseothricin retained 24.12 copies/cell (**Figure 5G**). Together, these results indicate that CEPT is essential for cell survival and proliferation during the promastigote stage of *L. major*.

### CEPT Is Dispensable for *L. major* Amastigotes

**Figure 6:**
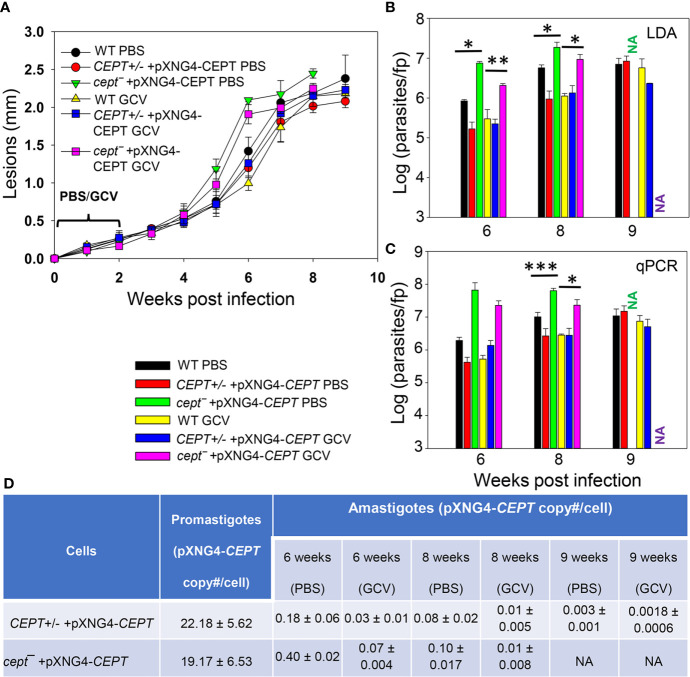
CEPT is dispensable for *L. major* amastigotes.** **BALB/c mice were infected in the footpads with stationary phase promastigotes and treated with either GCV or PBS from day 1 to day 14 post infection as described in *Materials and Methods*. **(A)** Footpad lesions were recorded weekly. **(B, C)** Parasite numbers in the infected footpads were determined at the indicated times by limiting dilution assay** (B)** and qPCR **(C)**. **(D)** The pXNG4-*CEPT* copy numbers in promastigotes and amastigotes (#/cell ± SDs) were determined by qPCR. The 9 weeks data for *cept*¯ +pXNG4-*CEPT* amastigotes were not available (NA) because the infected mice had reached the humane endpoint at 8 weeks. Error bars represent standard deviations (*p < 0.05, **p < 0.01, ***p < 0.001).

To investigate whether CEPT is required during the amastigote stage, we infected BALB/c mice in the footpad with WT, *CEPT+/−* +pXNG4*-CEPT*, or *cept¯+*pXNG4*-CEPT* promastigotes. Half of the infected mice received daily treatment of GCV for 14 days and the other half received equivalent amount of sterile PBS as controls. Parasite virulence was assessed by measuring the development of footpad lesions and parasite growth in mice was determined by limiting dilution assay (LDA) and qPCR analysis. No significant difference in body weight was detected between GCV and PBS treated mice (**Figure S2**). As expected, mice infected by WT parasites were not affected by GCV treatment in footpad lesion development (**Figure 6A**). Similar results were observed in mice infected by *CEPT+/−* +pXNG4*-CEPT* and *cept¯+*pXNG4*-CEPT* parasites as no significant differences were detected between the GCV- and PBS-treated groups (**Figure 6A**). We then determined parasite numbers in the footpads of infected mice at 6-, 8- and 9-weeks post infection (**Figures 6B, C**). While all groups showed robust parasite loads which increased over time, mice infected by *cept¯+*pXNG4*-CEPT* (both GCV- and PBS-treated groups) had more parasites than other groups at 6- and 8-weeks post infection (**Figures 6B, C**, 9-week data were not available for *cept¯+*pXNG4*-CEPT* because those mice had reached the humane endpoint after 8 weeks). These results were largely in agreement with the lesion development displayed by infected mice (**Figure 6A**). Thus, *cept¯+*pXNG4*-CEPT* parasites are fully virulent and proliferative in BALB/c mice even after GCV treatment.

Next, we examined whether *CEPT+/−* +pXNG4*-CEPT* and *cept¯+*pXNG4*-CEPT* parasites would retain the pXNG4*-CEPT* plasmid in mice. To do so, qPCR analyses were performed on the amastigote DNA isolated from infected mice at 6-, 8-, and 9-weeks post infection using primers for the pXNG4 plasmid (to determine plasmid copy number) and for the *L. major* 28S rRNA gene (to determine parasite number). The average plasmid copy number per cell was determined by dividing the total number of pXNG4 plasmid molecules with the total number of parasites. As illustrated in **Figure 6D**, *CEPT+/−* +pXNG4*-CEPT* amastigotes contained 0.08–0.18 copies of the episome per cell in PBS-treated mice at weeks 6–8 post infection, and GCV-treatment further reduced the average copy number to 0.01–0.03 per cell. At week 9 post infection, the pXNG4 copy number became nearly undetectable (<0.01, **Figure 6D**). Importantly, similar results were obtained for the *cept¯+*pXNG4*-CEPT* amastigotes as the episome copy numbers were 0.10−0.40 copies per cell in PBS-treated mice and 0.01−0.07 copies per cell in GCV-treated mice (**Figure 6D**). As controls, promastigotes of *CEPT+/−* +pXNG4*-CEPT* and *cept¯/+*pXNG4*-CEPT* cultivated in the presence of nourseothricin contained 19–24 copies of pXNG4*-CEPT* per cell, and those cultured in the presence of GCV contained 1.6–4.4 copies per cell (**Figures 6D** and **5G**). The fact that *cept¯+*pXNG4*-CEPT* amastigotes were fully virulent and replicative with very few copies of the episome (much less than one plasmid per cell) suggests that CEPT is dispensable during the mammalian (intracellular) phase. These findings are distinct from our previously reported *fpps¯+*pXNG4-*FPPS* parasites (Mukherjee et al., 2019). Thus, the *de novo* synthesis of PC is only required for the promastigote but not amastigote stage in *L major*.

Our conclusion was also supported by comparing the parasite loads data from LDA and qPCR (**Figures 6B, C**). For WT and *CEPT+/−* +pXNG4*-CEPT* parasites, results from LDA and qPCR were largely comparable (**Figures 6B, C**). In contrast, for *cept¯+*pXNG4*-CEPT*, the qPCR results were 4–10 times higher than the corresponding LDA results (2–6 × 10^7^ vs 2–20 × 10^6^, **Figure 6C**). While qPCR revealed the total number of amastigotes in the infected footpad, LDA only detected those promastigotes that had successfully converted from amastigotes and replicated *in vitro*. It appears that only a fraction of *cept¯+*pXNG4*-CEPT* amastigotes can be converted into proliferative promastigotes due to their low episome copy number (more on this point below).

### *Cept¯+*pXNG4*-CEPT* Parasites Regain the Plasmid After Conversion From Amastigotes to Promastigotes

**Figure 7:**
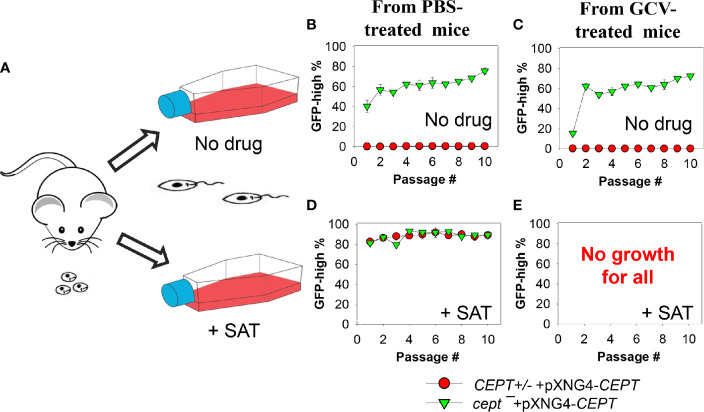
*Cept ¯*+pXNG4-*CEPT* parasites restore their episome levels after converting to promastigotes. **(A)** Amastigotes from infected BALB/c mice were allowed to recover in complete M199 media in the absence or presence of 200 µg/ml of nourseothricin (+ SAT). Promastigotes were then continuously cultivated in the absence **(B, C)** or presence **(D, E)** of nourseothricin for 10 passages and percentages of GFP-high cells were analyzed by flow cytometry for each passage. It is of note that no cells from GCV-treated mice were able to recover in nourseothricin **(E)**. For **(B–D)**, error bars represent standard deviations from two independent biological experiments (two technical duplicates each time).

The apparent lack of episome in *cept¯+*pXNG4*-CEPT* amastigotes (**Figure 6D**) prompted us to examine their ability to convert to promastigotes and proliferate in culture in more detail. First, *CEPT+/−* +pXNG4*-CEPT* and *cept¯+*pXNG4*-CEPT* amastigotes were isolated from infected mice (**Figure 6**) and allowed to recover in complete M199 media in the absence or presence of nourseothricin (“no drug” or “+ SAT” in **Figure 7A**). After amastigotes had converted into promastigotes (in 2–4 days), they were cultivated in the absence or presence of nourseothricin for 10 consecutive passages and their GFP fluorescence levels were determined by flow cytometry (**Figures 7B–E**). Without the positive selection drug nourseothricin, *cept¯+*pXNG4*-CEPT* parasites isolated from PBS-treated mice showed 40–60% GFP-high cells, whereas *CEPT+/−* +pXNG4*-CEPT* parasites lost most of the GFP-high cells (**Figure 7B**). Very similar results were observed for the parasites isolated from GCV-treated mice except that the GFP-high population in *cept¯+*pXNG4*-CEPT* was ~15% in the first passage before going up to 50–60% in the later passages (**Figure 7C**). The slower recovery may be due to the extremely low episome copy number in parasites isolated from in GCV-treated mice (0.01–0.07/cell for *cept¯+*pXNG4*-CEPT*, **Figure 6D**).

Meanwhile, if parasites were continuously cultivated in the presence of nourseothricin, both *cept¯+*pXNG4*-CEPT* and *CEPT+/−* +pXNG4*-CEPT* exhibited 80–90% GFP-high cells if they were isolated from PBS-treated mice (**Figure 7D**). However, parasites isolated from GCV-treated mice failed to grow in the presence of nourseothricin (**Figure 7E**). These results suggest that the extremely low episome levels in *CEPT+/−* +pXNG4*-CEPT* and *cept¯+*pXNG4*-CEPT* amastigotes isolated from GCV-treated mice (0.01−0.07 copies per cell, **Figure 6D**) prevented these parasites from converting into proliferative promastigotes in the presence of nourseothricin.

In addition, the effect of GCV on promastigote proliferation was examined (**Figure S3**). If *cept ^—^ +*pXNG4*-CEPT* amastigotes were allowed to convert into promastigotes in the absence of GCV (“no drug” in **Figure S3A**), no significant difference in replication was observed (**Figure S3B**). This was consistent with the rapid restoration of episome level in *cept ^—^ +*pXNG4*-CEPT* (**Figure 7**). However, if these amastigotes were forced to convert into promastigotes in the presence of GCV (“+ GCV” in **Figure S3A**), they showed a 24–96 h delay during the first passage (“P1” in **Figure S3B**). This delay was likely caused by the difficulty of these cells trying to increase their episome copy numbers to support growth while balancing the toxic effect from GCV. They were able to proliferate normally after the first passage (“P2-P4” in **Figure S3B**), suggesting adaptation to limiting the pXNG4 copy number to proper levels that satisfy the need of PC synthesis while limiting toxicity. We also analyzed the pXNG4*-CEPT* DNA from those recovered culture promastigotes (**Figure 7** and **Figure S3**) and did not detect any mutations in the plasmid based on restriction enzyme digestion and sequencing.

These findings suggest that when *cept¯* +pXNG4*-CEPT* amastigotes were allowed to recover during the initial passage in culture, they quickly elevated the plasmid copy numbers while transitioning to promastigotes. This occurred even in the absence of nourseothricin (the selection drug for pXNG4 plasmid) and presence of GCV due to the essentiality of CEPT in promastigotes. In contrast, for *CEPT+/−* +pXNG4*-CEPT*, the plasmid copy number did not increase without strong positive selection.

### *Cept¯+*pXNG4*-CEPT* Parasites Showed Similar PC and PE Contents as WT Parasites

Next, we determined the lipid composition in *cept ^—^ +*pXNG4*-CEPT* parasites to evaluate the impact of CEPT on PC/PE production. First, total lipids from culture promastigotes were examined by ESI-MS in the positive ion mode to detect PC (as protonated [M+H]^+^ ions) and negative ion mode to detect PE (as deprotonated [M-H]^-^ ions). As indicated in **Figure S4A**, the majority of PC in promastigotes were 1,2-diacyl types with polyunsaturated FAs at the sn-2 positions (e.g., 18:2/22:6 PC, 18:3/22:6 PC, and 16:1/20:4 PC) while ether PC (e.g., a18:0/20:2 PC and e18:1/24:6 PC) were less common. These findings were largely in agreement with previous reports (Weingartner et al., 2012; Williams et al., 2012). No statistically significant difference in PC composition was detected between WT and *cept¯+*pXNG4*-CEPT* promastigotes (**Figure S4A**). Similarly, the composition for PE (mainly e18:0/18:2 PE and e18:0/18:1 PE, both are PME) in *cept¯+*pXNG4*-CEPT* were very similar to WT promastigotes (**Figure S4B**) (Zhang et al., 2007).

**Figure 8:**
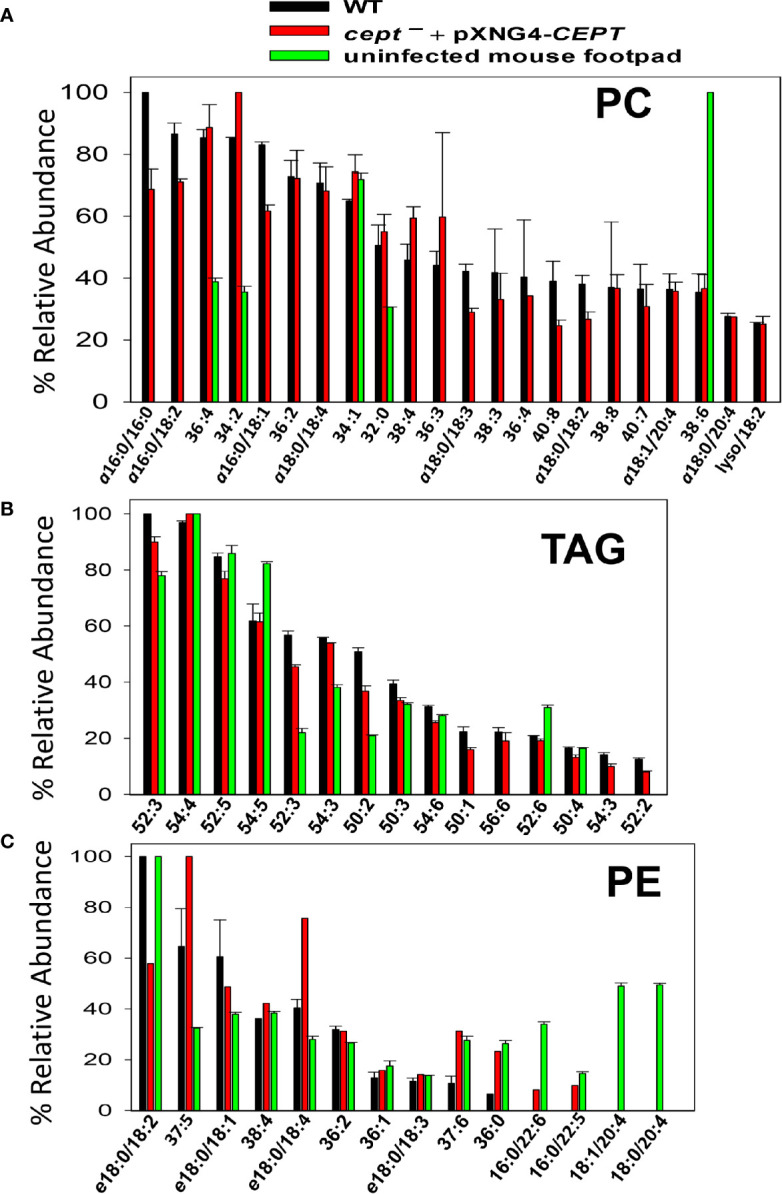
*Cept ¯*+pXNG4-*CEPT* amastigotes show similar composition of PC and PE as WT amastigotes. Amastigotes were isolated from the footpad of infected BALB/c mice. Lipids from partially purified amastigotes and uninfected mouse footpad tissue were analyzed by ESI-MS in the positive ion mode **(A, B)** and negative ion mode **(C)**. Compositions for PC **(A)**, TAG **(B)**, and PE **(C)** were summarized. Only major lipid species (>20% for PC and >10% for TAG and PE) by relative abundance are shown. Predicted formulas and molecular weights were indicated for each lipid species (*a*: 1-alkyl phospholipids, *e*: 1-alkenyl phospholipids, lyso: 1-lysophospholipids).

Finally, we examined the lipids from lesion-derived amastigotes (**Figure S5**) and uninfected mouse footpads. As summarized in **Figure 8A**, the PC in WT amastigotes included both ether lipids (e.g., a16:0/16:0 PC, a16:0/18:2 PC, and a16:0/18:1 PC) and 1,2-diacyl types (e.g., 36:4 PC, 36:2 PC, and 34:2 PC). Importantly, very similar PC composition was found in *cept¯+*pXNG4*-CEPT* amastigotes despite the near complete loss of *CEPT* gene in these parasites (**Figures 8A** and **6D**). Thus, *L. major* amastigotes can acquire the majority of their PC in the absence of *de novo* synthesis. While some of these amastigote PC (e.g., 36:4 PC, 34:2 PC, and 34:1 PC) were also found in uninfected mouse tissue, the majority of amastigote PC were of low abundance or undetectable in the host (**Figure 8A**) (e.g., a16:0/16:0 PC, a16:0/18:2 PC, and a16:0/18:1 PC). These findings suggest that amastigotes can generate PC through the uptake and remodeling of host PC.

In comparison to PC, we found higher degrees of overlap in the composition of triacylglycerol (TAG) and PE between amastigotes and mouse tissue (**Figures 8B, C**). Again, no major differences in TAG and PE composition were detected between WT and *cept¯+*pXNG4*-CEPT* amastigotes (**Figures 8B, C**). In summary, our data indicate that the *de novo* PC synthesis is dispensable for the survival and proliferation of intracellular amastigotes which can salvage and remodel lipids from the host.

## Discussion

*Leishmania* parasites can synthesize PC from multiple routes including the choline branch of the Kennedy pathway, the N-methylation of PE and the salvage/remodeling of host lipids (**Figure 1**). In this study, we characterized the gene encoding CEPT in *L. major* which catalyzes the formation of PC from CDP-choline and DAG, the final step of *de novo* PC synthesis (**Figures 1**, **4**). Similar to CPCT and EPT, CEPT is primarily located at the ER (**Figure 3**) (Pawlowic et al., 2016; Moitra et al., 2019). While both the choline branch and ethanolamine branch are essential for survival in *T. brucei* (Gibellini et al., 2009; Gibellini and Smith, 2010), *Leishmania* parasites can tolerate the loss of several Kennedy pathway enzymes presumably from the compensatory effects of other pathways, as evidenced by the *cpct¯* and *ept¯* mutants (Pawlowic et al., 2016; Moitra et al., 2019). However, we could not generate the chromosomal *CEPT* null mutant in culture without first introducing a complementing episome (**Figure 2**). Attempts to eliminate the GFP-containing pXNG4-*CEPT* episome *via* prolonged GCV-treatment in culture were unsuccessful in *cept¯*+pXNG4-*CEPT*, as 40–50% of parasites remained GFP-high (**Figure 5A**). Even after cell sorting and serial dilution, clones derived from the GFP-low population still displayed 31–35% GFP-high with 1.5–2 copies of pXNG4-*CEPT* episome per cell (**Figures 5D–G**). Thus, those GFP-low *cept¯*+pXNG4-*CEPT* parasites likely contained the pXNG4-*CEPT* episome at a low but detectable level or they could not proliferate without the GFP-high cells. Interestingly, *Leishmania* and *T. brucei* parasites are known to produce and exchange extracellular vesicles among individual cells (Douanne et al., 2020). Such transfer of cellular material may contribute to the survival of *cept¯*+pXNG4-*CEPT* parasites after GCV treatment.

Because CEPT is also expected to catalyze the synthesis of diacyl-PE, its deletion would not only block the choline branch of the Kennedy pathway, but also reduce PC synthesis from PE N-methylation (**Figure 1**). Therefore, while *L. major* promastigotes can withstand the deletion of CPCT, a complete loss of CEPT would drastically compromise their ability to generate PC. The essentiality of *CEPT* in promastigotes suggests that the salvage pathway by itself cannot produce enough PC (the most abundant lipids) to support parasite proliferation in culture. In our study, the complete M199 medium with 10% fetal bovine serum contains 20–30 µM of serine (which can be converted into EtN-P), 10–40 µM of choline, and 30–40 µM of total lipids (Kapler et al., 1990b; Zhang et al., 2007; Brunner et al., 2010). This culture condition would allow the proliferation of *cpct¯* and *ept¯* promastigotes although they showed significantly reduced growth under serum free or EtN-restrictive conditions (Pawlowic et al., 2016; Moitra et al., 2019). In sand flies, *Leishmania* promastigotes undergo two growth phases increasing cell numbers by 100-1,000-fold in 5–10 days (Gossage et al., 2003; Inbar et al., 2013). The availability of phospholipids in sand fly midgut after a blood meal is likely to be highly dynamic and difficult to assess, and we expect the *de novo* lipid synthesis to play a major role in supporting promastigote growth and development. This seems to correlate with the upregulation of fatty acid biosynthesis during *L. major* development in sand flies (Inbar et al., 2017).

In contrast to the promastigote stage, CEPT appears to be dispensable during the intracellular amastigote stage. In mice infected by *cept¯*+pXNG4-*CEPT* parasites, GCV treatment was able to dramatically reduce the episome level to 0.01–0.07 copy per cell after 6–8 weeks (**Figure 6D**). While this analysis was done at the DNA level, it suggests that the majority of *cept¯* +pXNG4-*CEPT* amastigotes lack CEPT mRNA and protein. Nonetheless, these chromosomal-null mutants were fully virulent and replicative in mice (**Figures 6A–C**). In fact, these *cept¯*+pXNG4-*CEPT* parasites showed slightly better growth than WT parasites in mice, suggesting that they gained a small advantage by losing *de novo* PC synthesis (**Figures 6B, C**). Additionally, lipidomic analysis revealed very similar PC contents between WT and *cept¯*+pXNG4-*CEPT* amastigotes (**Figure 8A**). The total PC levels in WT and *cept¯*+pXNG4-*CEPT* amastigotes also appeared to be similar based on the signal intensity from mass spectrometry. Thus, *L. major* amastigotes are capable of generating sufficient amounts of PC in the absence of *de novo* synthesis. This is consistent with the three-fold reduction of *CEPT* transcript level when parasites transition from promastigotes to amastigotes (**Figure 3A**).

In theory, amastigotes could acquire PC from the mammalian host, either by directly incorporating host lipids or converting them into their own through remodeling. As indicated in **Figure 8A**, while certain PC types were present in both amastigotes and mice, the majority of amastigote PC including the most abundant species like a16:0/16:0 PC and a16:0/18:2 PC were of low abundance or undetectable in uninfected mouse tissue, suggesting that they were modified from host lipids. The mechanism by which amastigotes remodel host lipids is under investigation. In many eukaryotes, phospholipase A2 (PLA2) removes fatty acids at the sn-2 position of PC, and the resulting lysophosphatidylcholine can be re-acylated at the sn-2 position to yield a different PC (Lands cycle) (Lands, 1958). Genes encoding PLA2 and lysophosphatidylcholine acyltransferase are present in *Leishmania* genomes suggesting that they can carry out lipid remodeling *via* deacylation/re-acylation. The Lands cycle is also present in the intestinal protozoan *Giardia lamblia* which has a limited capacity for *de novo* lipid synthesis (Das et al., 2001). In addition to PC remodeling and *de novo* synthesis, it is also possible for amastigotes to generate PC through the N-methylation of salvaged PE (**Figure 1**).

Comparing to PC, higher degrees of overlap were observed in the compositions of triacylglycerol (TAG) and PE between amastigotes and uninfected mouse tissue (**Figures 8B, C**). These findings support the direct incorporation of these lipids by amastigotes similar to the reported uptake of host TAG by intracellular *T. cruzi* parasites (Gazos-Lopes et al., 2017), although further investigation is warranted. Overall, the ability of *Leishmania* amastigotes to take up and remodel host PC is in agreement with their propensity to incorporate host sphingolipids and cholesterol (Zhang et al., 2005; Xu et al., 2014). Since *Leishmania* amastigotes spend the majority of their time in mammals in a slow growing, quiescent state (estimated doubling time: 60 h) (Saunders et al., 2014; Mandell and Beverley, 2017), the salvage pathway (which is less energy intensive than *de novo* synthesis) seems to fit the intracellular stage. In contrast, the *de novo* synthesis is required to generate sufficient amount of lipids to support parasite replication during the promastigote stage (estimated doubling time: 6–8 h in culture and 10–12 h in sand fly) (Gossage et al., 2003; Inbar et al., 2013). It is of interest to determine whether intracellular amastigotes require any *de novo* synthesis to generate certain parasite-specific lipids that cannot be acquired from the host or to complement the salvage pathway. It is also important to explore the PC/PE synthesis in *Leishmania* species besides *L. major*. While the composition of PC/PE appears to be largely conserved across several *Leishmania* species (Wassef et al., 1985; Ramos et al., 2008; Pulido et al., 2017), different parasites may acquire lipids differently which may affect their interaction with the host and their tissue tropism. The ability of *Leishmania* parasites to acquire lipids *via* either *de novo* synthesis or uptake/remodeling (the metabolic flexibility) may allow them to adapt to a diverse range of host cells and host species.

Finally, while the *de novo* synthesis of PC appears to be dispensable for *L. major* amastigotes, losing CPCT or CEPT would make them highly dependent on salvage or other pathways to acquire PC. Thus, inhibitors of phospholipids uptake or PE N-methylation may yield enhanced efficacy when used in combination with *de novo* synthesis blockers. Intriguingly, treatment of *L. donovani* promastigotes with miltefosine, a lysophosphatidylcholine analog, reduced the PC content and enhanced the PE level, suggesting a partial inactivation of PE N-methyltransferase (Rakotomanga et al., 2007). In addition to its impact on PC synthesis, miltefosine also compromise the function of parasite mitochondrion and the intracellular calcium homeostasis (Paris et al., 2004; Luque-Ortega and Rivas, 2007; Pinto-Martinez et al., 2018), so using it in conjunction with inhibitors that affect the same targets may produce synergistic effects. Future studies will also elucidate how amastigotes regulate the uptake and remodeling of different classes of lipids and explore the potential of blocking the transfer of host lipids to parasites as a novel therapeutic.

## Data Availability Statement

The raw data supporting the conclusions of this article will be made available by the authors, without undue reservation.

## Ethics Statement

The animal study was reviewed and approved by Texas Tech University Animal Care and Use Committee.

## Author Contributions

SM, SB, MP, and F-fH performed the experiments and analyzed the data. SM and KZ wrote the manuscript. KZ and F-fH acquired the funding. All authors contributed to the article and approved the submitted version.

## Funding

This work was supported by the US National Institutes of Health grants AI099380 (KZ), P41-GM103422 (FH), P60-DK20579 (FH), and P30-DK56341 (FH) for the Biomedical Mass Spectrometry Resource at Washington University in St. Louis, MO, USA). The funder had no role in study design, data collection and analysis, decision to publish, or preparation of the manuscript.

## Conflict of Interest

The authors declare that the research was conducted in the absence of any commercial or financial relationships that could be construed as a potential conflict of interest.

## References

[B1] Alvar J. Velez I. D. Bern C. Herrero M. Desjeux P. Cano J.. (2012). Leishmaniasis worldwide and global estimates of its incidence. PLoS One 7 (5), e35671. 10.1371/journal.pone.0035671 22693548 PMC3365071

[B2] Beach D. H. Holz G. G. Jr. Anekwe G. E. (1979). Lipids of Leishmania promastigotes. J. Parasitol. 65 (2), 201–216. 10.2307/3280147 448607

[B3] Besteiro S. Williams R. A. Morrison L. S. Coombs G. H. Mottram J. C. (2006). Endosome sorting and autophagy are essential for differentiation and virulence of *Leishmania major* . J. Biol. Chem. 281 (16), 11384–11396. 10.1074/jbc.M512307200 16497676

[B4] Bibis S. S. Dahlstrom K. Zhu T. Zufferey R. (2014). Characterization of Leishmania major phosphatidylethanolamine methyltransferases LmjPEM1 and LmjPEM2 and their inhibition by choline analogs. Mol. Biochem. Parasitol. 196 (2), 90–99. 10.1016/j.molbiopara.2014.08.005 25176160 PMC4252796

[B5] Bligh E. G. Dyer W. J. (1959). A rapid method of total lipid extraction and purification. Can. J. Biochem. Physiol. 37 (8), 911–917. 10.1139/o59-099 13671378

[B6] Bolte S. Cordelieres F. P. (2006). A guided tour into subcellular colocalization analysis in light microscopy. J. Microsc. 224 (Pt 3), 213–232. 10.1111/j.1365-2818.2006.01706.x 17210054

[B7] Brunner D. Frank J. Appl H. Schoffl H. Pfaller W. Gstraunthaler G. (2010). Serum-free cell culture: the serum-free media interactive online database. ALTEX 27 (1), 53–62. 10.14573/altex.2010.1.53 20390239

[B8] Castanys-Munoz E. Alder-Baerens N. Pomorski T. Gamarro F. Castanys S. (2007). A novel ATP-binding cassette transporter from Leishmania is involved in transport of phosphatidylcholine analogues and resistance to alkyl-phospholipids. Mol. Microbiol. 64 (5), 1141–1153. 10.1111/j.1365-2958.2007.05653.x 17542911

[B9] Croft S. L. Olliaro P. (2011). Leishmaniasis chemotherapy–challenges and opportunities. Clin. Microbiol. Infect. 17 (10), 1478–1483. 10.1111/j.1469-0691.2011.03630.x 21933306

[B10] Cruz A. Coburn C. M. Beverley S. M. (1991). Double targeted gene replacement for creating null mutants. Proc. Natl. Acad. Sci. U. S. A. 88 (16), 7170–7174. 10.1073/pnas.88.16.7170 1651496 PMC52255

[B11] Cui Z. Vance D. E. (1996). Expression of phosphatidylethanolamine N-methyltransferase-2 is markedly enhanced in long term choline-deficient rats. J. Biol. Chem. 271 (5), 2839–2843. 10.1074/jbc.271.5.2839 8576263

[B12] Das S. Castillo C. Stevens T. (2001). Phospholipid remodeling/generation in Giardia: the role of the Lands cycle. Trends Parasitol. 17 (7), 316–319. 10.1016/S1471-4922(01)01901-8 11423372

[B13] Douanne N. Dong G. Douanne M. Olivier M. Fernandez-Prada C. (2020). Unravelling the proteomic signature of extracellular vesicles released by drug-resistant Leishmania infantum parasites. PLoS Negl. Trop. Dis. 14 (7), e0008439. 10.1371/journal.pntd.0008439 32628683 PMC7365475

[B14] Ellens H. Siegel D. P. Alford D. Yeagle P. L. Boni L. Lis L. J.. (1989). Membrane fusion and inverted phases. Biochemistry 28 (9), 3692–3703. 10.1021/bi00435a011 2751990

[B15] Exton J. H. (1994). Phosphatidylcholine breakdown and signal transduction. Biochim. Biophys. Acta 1212 (1), 26–42. 10.1016/0005-2760(94)90186-4 8155724

[B16] Farine L. Niemann M. Schneider A. Butikofer P. (2015). Phosphatidylethanolamine and phosphatidylcholine biosynthesis by the Kennedy pathway occurs at different sites in Trypanosoma brucei. Sci. Rep. 5, 16787. 10.1038/srep16787 26577437 PMC4649479

[B17] Furse S. de Kroon A. I. (2015). Phosphatidylcholine’s functions beyond that of a membrane brick. Mol. Membr. Biol. 32 (4), 117–119. 10.3109/09687688.2015.1066894 26306852

[B18] Gazos-Lopes F. Martin J. L. Dumoulin P. C. Burleigh B. A. (2017). Host triacylglycerols shape the lipidome of intracellular trypanosomes and modulate their growth. PLoS Pathog. 13 (12), e1006800. 10.1371/journal.ppat.1006800 29281741 PMC5760102

[B20] Gibellini F. Smith T. K. (2010). The Kennedy pathway–De novo synthesis of phosphatidylethanolamine and phosphatidylcholine. IUBMB Life 62 (6), 414–428. 10.1002/iub.337 20503434

[B19] Gibellini F. Hunter W. N. Smith T. K. (2009). The ethanolamine branch of the Kennedy pathway is essential in the bloodstream form of Trypanosoma brucei. Mol. Microbiol. 73 (5), 826–843. 10.1111/j.1365-2958.2009.06764.x 19555461 PMC2784872

[B21] Glaser T. A. Wells S. J. Spithill T. W. Pettitt J. M. Humphris D. C. Mukkada A. J. (1990). Leishmania major and L. donovani: a method for rapid purification of amastigotes. Exp. Parasitol. 71 (3), 343–345. 10.1016/0014-4894(90)90039-f 2209791

[B22] Gossage S. M. Rogers M. E. Bates P. A. (2003). Two separate growth phases during the development of Leishmania in sand flies: implications for understanding the life cycle. Int. J. Parasitol. 33 (10), 1027–1034. 10.1016/S0020-7519(03)00142-5 13129524 PMC2839921

[B23] Ha D. S. Schwarz J. K. Turco S. J. Beverley S. M. (1996). Use of the green fluorescent protein as a marker in transfected Leishmania. Mol. Biochem. Parasitol. 77 (1), 57–64. 10.1016/0166-6851(96)02580-7 8784772

[B24] Henneberry A. L. McMaster C. R. (1999). Cloning and expression of a human choline/ethanolaminephosphotransferase: synthesis of phosphatidylcholine and phosphatidylethanolamine. Biochem. J. 339 ( Pt 2), 291–298. 10191259 PMC1220157

[B25] Henriques C. Atella G. C. Bonilha V. L. de Souza W. (2003). Biochemical analysis of proteins and lipids found in parasitophorous vacuoles containing Leishmania amazonensis. Parasitol. Res. 89 (2), 123–133. 10.1007/s00436-002-0728-y 12489012

[B26] Hsu F. F. Kuhlmann F. M. Turk J. Beverley S. M. (2014). Multiple-stage linear ion-trap with high resolution mass spectrometry towards complete structural characterization of phosphatidylethanolamines containing cyclopropane fatty acyl chain in Leishmania infantum. J. Mass Spectrom. 49 (3), 201–209. 10.1002/jms.3327 24619546 PMC4007172

[B27] Inbar E. Akopyants N. S. Charmoy M. Romano A. Lawyer P. Elnaiem D. E.. (2013). The mating competence of geographically diverse Leishmania major strains in their natural and unnatural sand fly vectors. PLoS Genet. 9 (7), e1003672. 10.1371/journal.pgen.1003672 23935521 PMC3723561

[B28] Inbar E. Hughitt V. K. Dillon L. A. Ghosh K. El-Sayed N. M. Sacks D. L. (2017). The Transcriptome of Leishmania major Developmental Stages in Their Natural Sand Fly Vector. MBio 8 (2), e00029-17. 10.1128/mBio.00029-17 28377524 PMC5380837

[B29] Kapler G. M. Coburn C. M. Beverley S. M. (1990a). Stable transfection of the human parasite *Leishmania major* delineates a 30-kilobase region sufficient for extrachromosomal replication and expression. Mol. Cell Biol. 10 (3), 1084–1094. 10.1128/MCB.10.3.1084 2304458 PMC360971

[B30] Kapler G. M. Zhang K. Beverley S. M. (1990b). Nuclease mapping and DNA sequence analysis of transcripts from the dihydrofolate reductase-thymidylate synthase (R) region of Leishmania major. Nucleic Acids Res. 18 (21), 6399–6408. 10.1093/nar/18.21.6399 2243782 PMC332520

[B31] Kennedy E. P. (1956). The synthesis of cytidine diphosphate choline, cytidine diphosphate ethanolamine, and related compounds. J. Biol. Chem. 222 (1), 185–191. 10.1016/S0021-9258(19)50784-0 13366992

[B32] Lands W. E. (1958). Metabolism of glycerolipides; a comparison of lecithin and triglyceride synthesis. J. Biol. Chem. 231 (2), 883–888. 10.1016/S0021-9258(18)70453-5 13539023

[B33] Livak K. J. Schmittgen T. D. (2001). Analysis of relative gene expression data using real-time quantitative PCR and the 2(-Delta Delta C(T)) Method. Methods 25 (4), 402–408. 10.1006/meth.2001.1262 11846609

[B34] Luque-Ortega J. R. Rivas L. (2007). Miltefosine (hexadecylphosphocholine) inhibits cytochrome c oxidase in Leishmania donovani promastigotes. Antimicrob. Agents Chemother. 51 (4), 1327–1332. 10.1128/AAC.01415-06 17283192 PMC1855476

[B35] Mandell M. A. Beverley S. M. (2017). Continual renewal and replication of persistent Leishmania major parasites in concomitantly immune hosts. Proc. Natl. Acad. Sci. U. S. A. 114 (5), E801–E810. 10.1073/pnas.1619265114 28096392 PMC5293024

[B36] Menon A. K. Eppinger M. Mayor S. Schwarz R. T. (1993). Phosphatidylethanolamine is the donor of the terminal phosphoethanolamine group in trypanosome glycosylphosphatidylinositols. EMBO J. 12 (5), 1907–1914. 10.1002/j.1460-2075.1993.tb05839.x 8491183 PMC413411

[B37] Moitra S. Pawlowic M. C. Hsu F. F. Zhang K. (2019). Phosphatidylcholine synthesis through cholinephosphate cytidylyltransferase is dispensable in Leishmania major. Sci. Rep. 9 (1), 7602. 10.1038/s41598-019-44086-6 31110206 PMC6527706

[B38] Mukherjee S. Basu S. Zhang K. (2019). Farnesyl pyrophosphate synthase is essential for the promastigote and amastigote stages in Leishmania major. Mol. Biochem. Parasitol. 230, 8–15. 10.1016/j.molbiopara.2019.03.001 30926449 PMC6529949

[B39] Murta S. M. Vickers T. J. Scott D. A. Beverley S. M. (2009). Methylene tetrahydrofolate dehydrogenase/cyclohydrolase and the synthesis of 10-CHO-THF are essential in Leishmania major. Mol. Microbiol. 71 (6), 1386–1401. 10.1111/j.1365-2958.2009.06610.x 19183277 PMC2692627

[B40] Nickels J. D. Smith J. C. Cheng X. (2015). Lateral organization, bilayer asymmetry, and inter-leaflet coupling of biological membranes. Chem. Phys. Lipids 192, 87–99. 10.1016/j.chemphyslip.2015.07.012 26232661

[B41] Paris C. Loiseau P. M. Bories C. Breard J. (2004). Miltefosine induces apoptosis-like death in Leishmania donovani promastigotes. Antimicrob. Agents Chemother. 48 (3), 852–859. 10.1128/AAC.48.3.852-859.2004 14982775 PMC353131

[B42] Parodi-Talice A. Araujo J. M. Torres C. Perez-Victoria J. M. Gamarro F. Castanys S. (2003). The overexpression of a new ABC transporter in Leishmania is related to phospholipid trafficking and reduced infectivity. Biochim. Biophys. Acta 1612 (2), 195–207. 10.1016/S0005-2736(03)00131-7 12787938

[B44] Pawlowic M. C. Zhang K. (2012). Leishmania parasites possess a platelet-activating factor acetylhydrolase important for virulence. Mol. Biochem. Parasitol. 186 (1), 11–20. 10.1016/j.molbiopara.2012.08.005 22954769 PMC3492548

[B43] Pawlowic M. C. Hsu F. F. Moitra S. Biyani N. Zhang K. (2016). Plasmenylethanolamine synthesis in Leishmania major. Mol. Microbiol. 101 (2), 238–249. 10.1111/mmi.13387 27062077 PMC4935589

[B45] Pinto-Martinez A. K. Rodriguez-Duran J. Serrano-Martin X. Hernandez-Rodriguez V. Benaim G. (2018). Mechanism of Action of Miltefosine on Leishmania donovani Involves the Impairment of Acidocalcisome Function and the Activation of the Sphingosine-Dependent Plasma Membrane Ca(2+) Channel. Antimicrob. Agents Chemother. 62 (1), e01614–17. 10.1128/AAC.01614-17 29061745 PMC5740361

[B46] Pulido S. A. Nguyen V. H. Alzate J. F. Cedeno D. L. Makurath M. A. Rios-Vasquez A.. (2017). Insights into the phosphatidylcholine and phosphatidylethanolamine biosynthetic pathways in Leishmania parasites and characterization of a choline kinase from Leishmania infantum. Comp. Biochem. Physiol. B Biochem. Mol. Biol. 213, 45–54. 10.1016/j.cbpb.2017.07.008 28754315

[B47] Rakotomanga M. Blanc S. Gaudin K. Chaminade P. Loiseau P. M. (2007). Miltefosine affects lipid metabolism in Leishmania donovani promastigotes. Antimicrob. Agents Chemother. 51 (4), 1425–1430. 10.1128/AAC.01123-06 17242145 PMC1855451

[B48] Ramos R. G. Libong D. Rakotomanga M. Gaudin K. Loiseau P. M. Chaminade P. (2008). Comparison between charged aerosol detection and light scattering detection for the analysis of Leishmania membrane phospholipids. J. Chromatogr. A 1209 (1-2), 88–94. 10.1016/j.chroma.2008.07.080 18823632

[B49] Saunders E. C. Ng W. W. Kloehn J. Chambers J. M. Ng M. McConville M. J. (2014). Induction of a stringent metabolic response in intracellular stages of Leishmania mexicana leads to increased dependence on mitochondrial metabolism. PLoS Pathog. 10 (1), e1003888. 10.1371/journal.ppat.1003888 24465208 PMC3900632

[B51] Signorell A. Jelk J. Rauch M. Butikofer P. (2008a). Phosphatidylethanolamine is the precursor of the ethanolamine phosphoglycerol moiety bound to eukaryotic elongation factor 1A. J. Biol. Chem. 283 (29), 20320–20329. 10.1074/jbc.M802430200 18499667

[B52] Signorell A. Rauch M. Jelk J. Ferguson M. A. Butikofer P. (2008b). Phosphatidylethanolamine in Trypanosoma brucei is organized in two separate pools and is synthesized exclusively by the Kennedy pathway. J. Biol. Chem. 283 (35), 23636–23644. 10.1074/jbc.M803600200 18587155 PMC3259767

[B50] Signorell A. Gluenz E. Rettig J. Schneider A. Shaw M. K. Gull K.. (2009). Perturbation of phosphatidylethanolamine synthesis affects mitochondrial morphology and cell-cycle progression in procyclic-form Trypanosoma brucei. Mol. Microbiol. 72 (4), 1068–1079. 10.1111/j.1365-2958.2009.06713.x 19400804

[B53] Smith T. K. Butikofer P. (2010). Lipid metabolism in Trypanosoma brucei. Mol. Biochem. Parasitol. 172 (2), 66–79. 10.1016/j.molbiopara.2010.04.001 20382188 PMC3744938

[B54] Titus R. G. Marchand M. Boon T. Louis J. (1985). A limiting dilution assay for quantifying Leishmania major in tissues of infected mice. Parasite Immunol. 7 (5), 545–555. 10.1111/j.1365-3024.1985.tb00098.x 3877902

[B55] van Meer G. Voelker D. R. Feigenson G. W. (2008). Membrane lipids: where they are and how they behave. Nat. Rev. Mol. Cell Biol. 9 (2), 112–124. 10.1038/nrm2330 18216768 PMC2642958

[B56] Vance J. E. (2008). Phosphatidylserine and phosphatidylethanolamine in mammalian cells: two metabolically related aminophospholipids. J. Lipid Res. 49 (7), 1377–1387. 10.1194/jlr.R700020-JLR200 18204094

[B57] Verkleij A. J. Leunissen-Bijvelt J. de Kruijff B. Hope M. Cullis P. R. (1984). Non-bilayer structures in membrane fusion. Ciba Found. Symp. 103, 45–59. 10.1002/9780470720844.ch4 6561137

[B58] Wassef M. K. Fioretti T. B. Dwyer D. M. (1985). Lipid analyses of isolated surface membranes of Leishmania donovani promastigotes. Lipids 20 (2), 108–115. 10.1007/BF02534216 3982233

[B59] Weingartner A. Kemmer G. Muller F. D. Zampieri R. A. Gonzaga dos Santos M. Schiller J.. (2012). Leishmania promastigotes lack phosphatidylserine but bind annexin V upon permeabilization or miltefosine treatment. PLoS One 7 (8), e42070. 10.1371/journal.pone.0042070 22870283 PMC3411662

[B60] Williams R. A. Smith T. K. Cull B. Mottram J. C. Coombs G. H. (2012). ATG5 is essential for ATG8-dependent autophagy and mitochondrial homeostasis in Leishmania major. PLoS Pathog. 8 (5), e1002695. 10.1371/journal.ppat.1002695 22615560 PMC3355087

[B61] Xu W. Hsu F. F. Baykal E. Huang J. Zhang K. (2014). Sterol Biosynthesis Is Required for Heat Resistance but Not Extracellular Survival in Leishmania. PLoS Pathog. 10 (10), e1004427. 10.1371/journal.ppat.1004427 25340392 PMC4207814

[B62] Zachowski A. (1993). Phospholipids in animal eukaryotic membranes: transverse asymmetry and movement. Biochem. J. 294 ( Pt 1), 1–14. 10.1042/bj2940001 8363559 PMC1134557

[B63] Zhang K. Beverley S. M. (2010). Phospholipid and sphingolipid metabolism in Leishmania. Mol. Biochem. Parasitol. 170 (2), 55–64. 10.1016/j.molbiopara.2009.12.004 20026359 PMC2815228

[B64] Zhang K. Hsu F. F. Scott D. A. Docampo R. Turk J. Beverley S. M. (2005). *Leishmania* salvage and remodelling of host sphingolipids in amastigote survival and acidocalcisome biogenesis. Mol. Microbiol. 55 (5), 1566–1578. 10.1111/j.1365-2958.2005.04493.x 15720561 PMC3803142

[B65] Zhang K. Pompey J. M. Hsu F. F. Key P. Bandhuvula P. Saba J. D.. (2007). Redirection of sphingolipid metabolism toward *de novo* synthesis of ethanolamine in *Leishmania* . EMBO J. 26 (4), 1094–1104. 10.1038/sj.emboj.7601565 17290222 PMC1852826

[B66] Zhang O. Xu W. Pillai A. Zhang K. (2012). Developmentally Regulated Sphingolipid Degradation in *Leishmania major* . PLoS One 7 (1), e31059. 10.1371/journal.pone.0031059 22299050 PMC3267774

[B67] Zheng L. T’Kind R. Decuypere S. von Freyend S. J. Coombs G. H. Watson D. G. (2010). Profiling of lipids in Leishmania donovani using hydrophilic interaction chromatography in combination with Fourier transform mass spectrometry. Rapid Commun. Mass Spectrom. 24 (14), 2074–2082. 10.1002/rcm.4618 20552712

[B68] Zufferey R. Allen S. Barron T. Sullivan D. R. Denny P. W. Almeida I. C.. (2003). Ether phospholipids and glycosylinositolphospholipids are not required for amastigote virulence or for inhibition of macrophage activation by *Leishmania major* . J. Biol. Chem. 278 (45), 44708–44718. 10.1074/jbc.M308063200 12944391

