# Supplementary material for: De Novo Synthesis of Phosphatidylcholine Is Essential for the Promastigote But Not Amastigote Stage in Leishmania major
Source: Front Cell Infect Microbiol. 2021 Mar 12;11:647870. doi: 10.3389/fcimb.2021.647870 (PMC7996062; doi:10.3389/fcimb.2021.647870)
Supplement: Supplementary file 1 [file DataSheet_1.pdf]

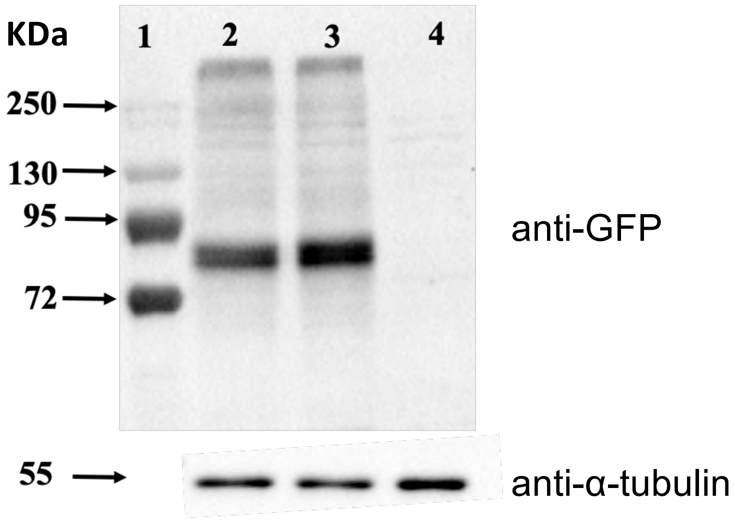

Figure S1. Validation of GFP-tagged CEPT expression by Western blot. Cell lysates from log phase *CEPT*+/−+pXG-*GFP-CEPT* clone 1 and 2 (Lane 2 and 3 respectively) and LV39WT promastigotes (lane 4) were analyzed by Western blot using antibodies against GFP (top) or α-tubulin (bottom). Lane 1: molecular weight marker.
