# Supplementary material for: De Novo Synthesis of Phosphatidylcholine Is Essential for the Promastigote But Not Amastigote Stage in Leishmania major
Source: Front Cell Infect Microbiol. 2021 Mar 12;11:647870. doi: 10.3389/fcimb.2021.647870 (PMC7996062; doi:10.3389/fcimb.2021.647870)
Supplement: Supplementary file 2 [file DataSheet_2.pdf]

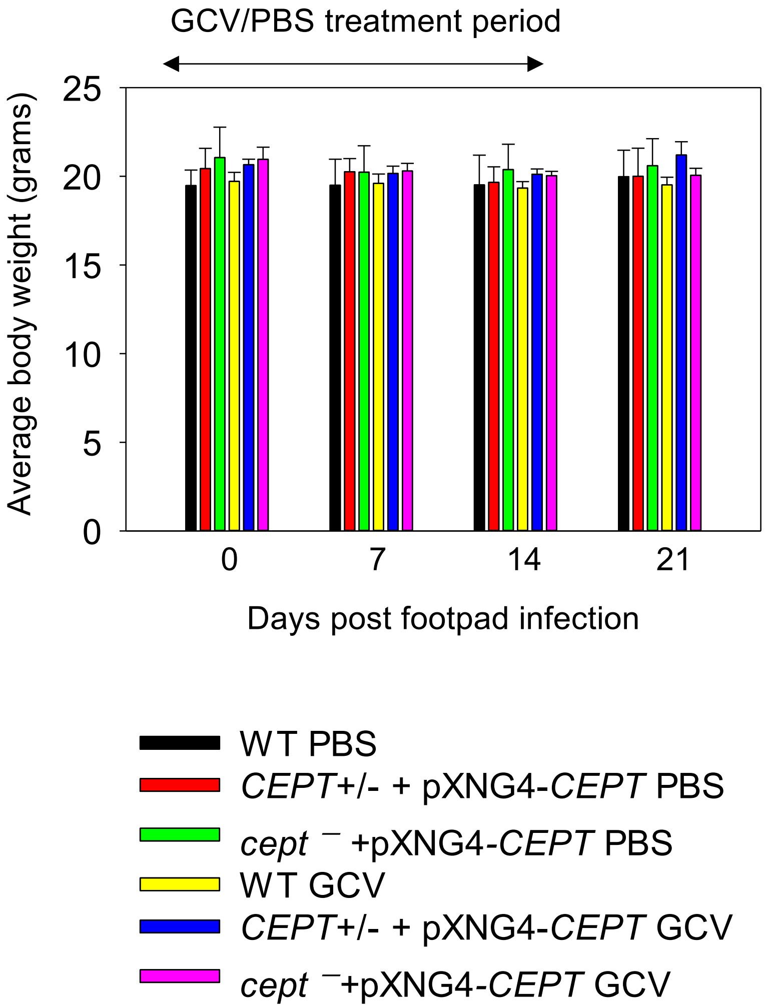

Figure S2. GCV treatment did not affect the body weights of infected BALB/c mice. Following infection with stationary phase promastigotes, half of the mice were treated daily with GCV for 14 days and the other group were treated with PBS. Mouse body weights were measured once a week for 3 weeks post infection. Error bars represent standard deviations (5 mice per group).
