# Supplementary material for: De Novo Synthesis of Phosphatidylcholine Is Essential for the Promastigote But Not Amastigote Stage in Leishmania major
Source: Front Cell Infect Microbiol. 2021 Mar 12;11:647870. doi: 10.3389/fcimb.2021.647870 (PMC7996062; doi:10.3389/fcimb.2021.647870)
Supplement: Supplementary file 3 [file DataSheet_3.pdf]

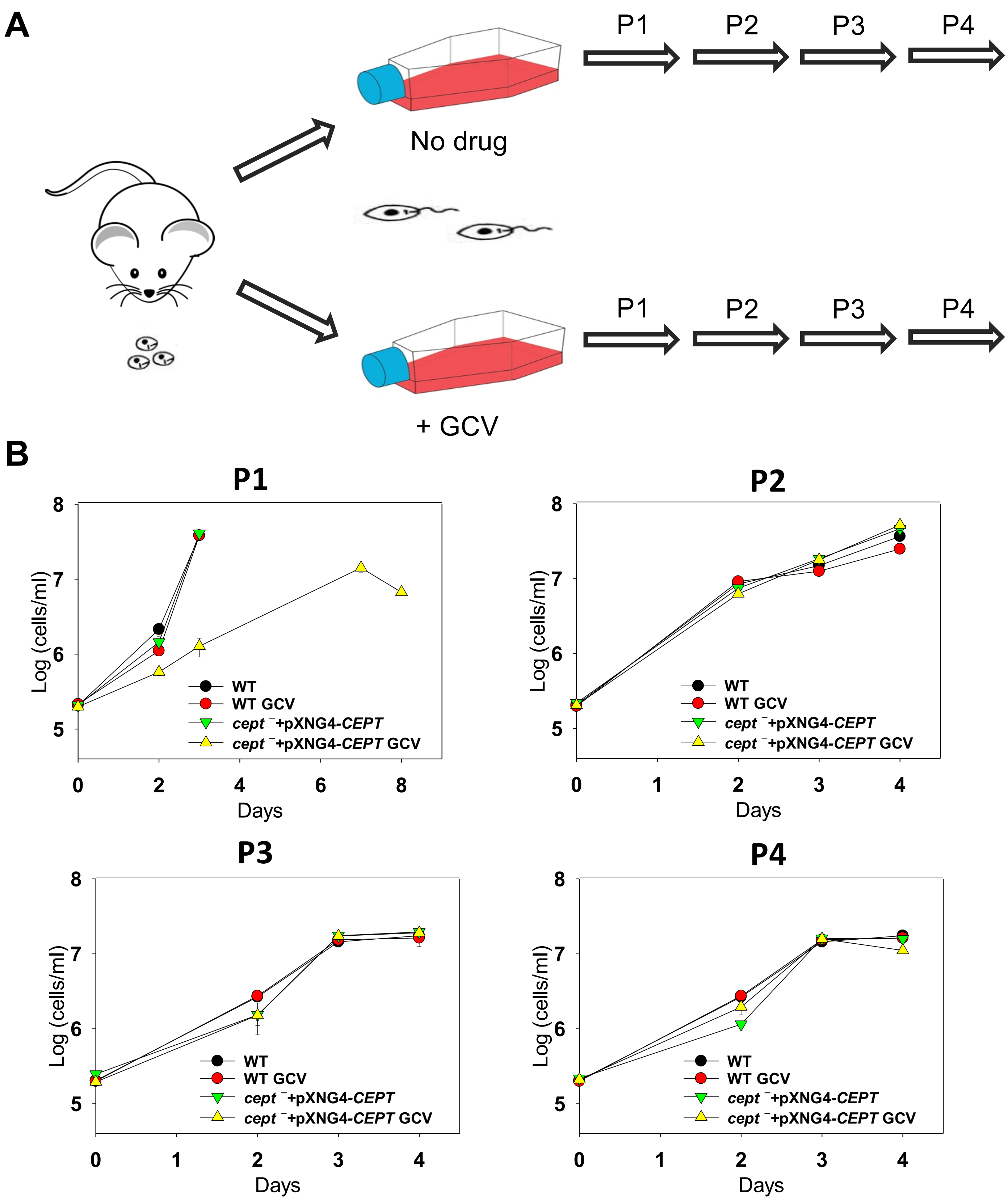

Figure S3. GCV treatment delayed the growth of *cept*⁻+pXNG4-*CEPT* promastigotes in the initial passage after recovery from mice. (**A**) Experimental scheme. Amastigotes were isolated from mice and allowed to recover in complete M199 media in the absence or presence 50 µg/ml of GCV. (**B**) In subsequent passages (P1-P4), promastigotes were inoculated at 2.0 x 10^5 cells/ml in the absence or presence 50 µg/ml of GCV. For each passage, culture densities were determined daily.
