# Supplementary material for: De Novo Synthesis of Phosphatidylcholine Is Essential for the Promastigote But Not Amastigote Stage in Leishmania major
Source: Front Cell Infect Microbiol. 2021 Mar 12;11:647870. doi: 10.3389/fcimb.2021.647870 (PMC7996062; doi:10.3389/fcimb.2021.647870)
Supplement: Supplementary file 4 [file DataSheet_4.pdf]

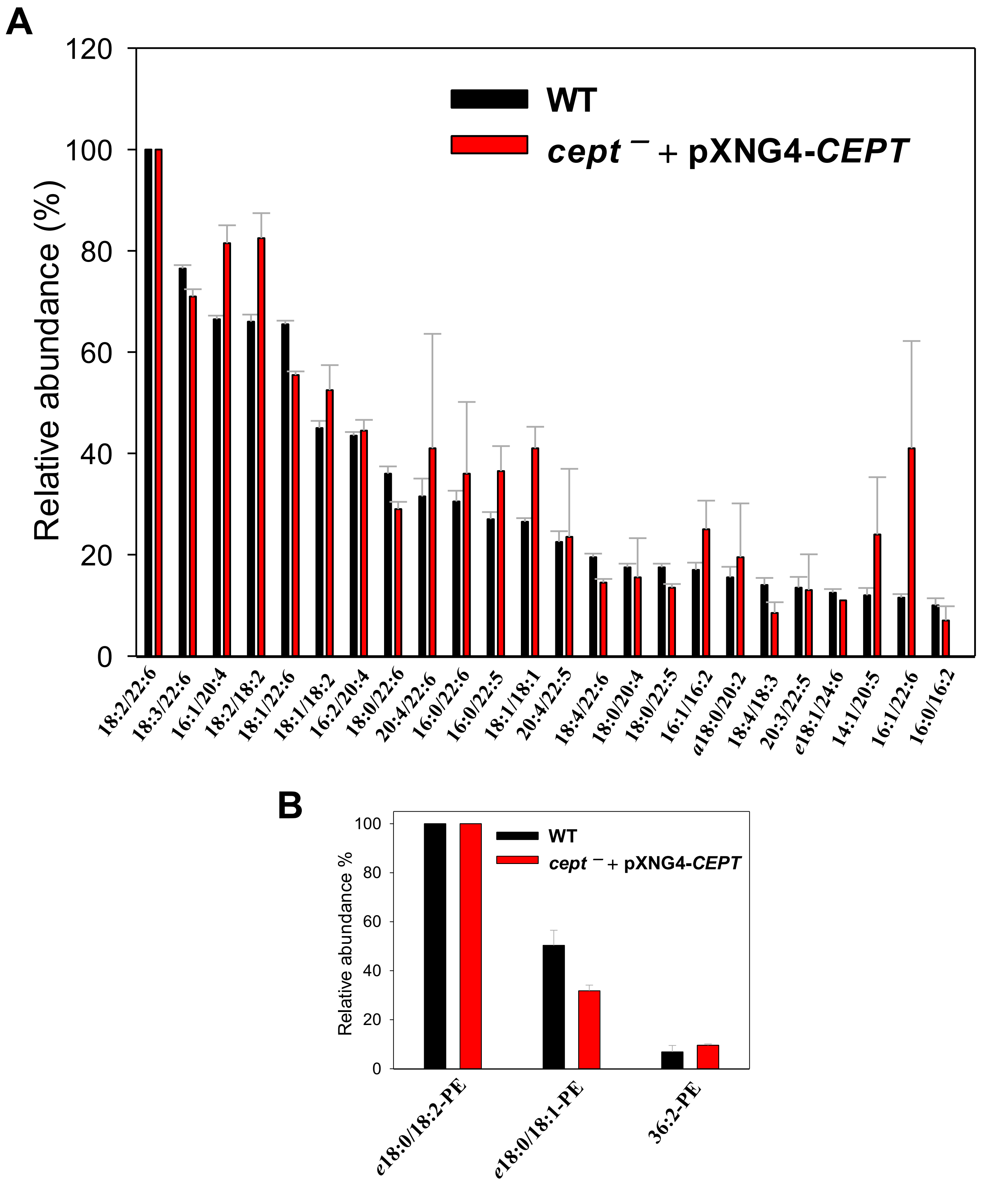

Figure S4. ***Cept*** **⁻+pXNG4-*CEPT* promastigotes show similar phospholipid composition as WT promastigotes.** Lipids from log phase promastigotes were analyzed by ESI-MS in the positive ion mode (**A**: for PC) and negative ion mode (**B**: for PE). Predicted fatty acyl constituents were indicated. Only major PC and PE species (>5% by relative abundance) are shown.
