# Supplementary material for: De Novo Synthesis of Phosphatidylcholine Is Essential for the Promastigote But Not Amastigote Stage in Leishmania major
Source: Front Cell Infect Microbiol. 2021 Mar 12;11:647870. doi: 10.3389/fcimb.2021.647870 (PMC7996062; doi:10.3389/fcimb.2021.647870)
Supplement: Supplementary file 5 [file DataSheet_5.pdf]

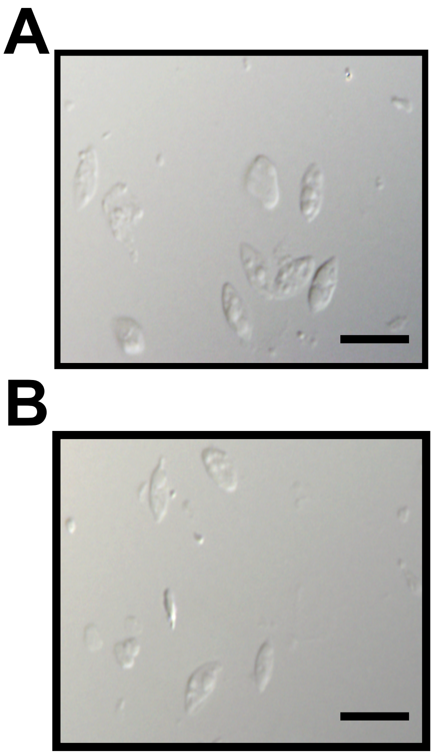

Figure S5. DIC images of partially purified amastigotes. Amastigotes of WT (**A**) and *cept* $^{-}$+pXNG4-*CEPT* (**B**) were isolated from infected BALB/c mice and partially purified as described in Materials and Methods. Scale bars: 10 µm.
