# Supplementary material for: De Novo Synthesis of Phosphatidylcholine Is Essential for the Promastigote But Not Amastigote Stage in Leishmania major
Source: Front Cell Infect Microbiol. 2021 Mar 12;11:647870. doi: 10.3389/fcimb.2021.647870 (PMC7996062; doi:10.3389/fcimb.2021.647870)
Supplement: Supplementary file 6 [file DataSheet_6.pdf]

Table S1. List of oligonucleotides used in this study

Primer #	Primer name	Sequence
#129	5’ *CEPT* ORF	GATCAGGGATCCACCATGCCCCCGAAGTCGATGGC
#130	3’ *CEPT* ORF	GATCATGGATCCCTAATCTGACTTATTCGGTT
#137	*CEPT* 5’ UTR Upstream	GATCATGAATTCGACGGAACTCTTAGCCACTC
#138	*CEPT* 5’ UTR Downstream	GTCAGCGGATCCGATCTAACTAGTCTTCGTTCTCCTTGTTTTGG
#139	*CEPT* 3’ UTR Upstream	GATCATGGATCCAGCAGCTGCGAAGTCCGCCC
#140	*CEPT* 3’ UTR Downstream	GATCATAAGCTTTACACCACCTCCTCGTCAAG
#784	C*EPT* qRT forward	GGAGAGTATCAACCCGCTCG
#785	*CEPT* qRT reverse	CAGTACCGCTGCAGGACATA
#780	28S rRNA gene forward	AAGATGGACCGGCCTCTAGT
#781	28S rRNA gene reverse	ATCCTTCCCCGCTCCAGTAT
#782	pXNG4 forward	CCCGACAACCACTACCTGAG
#783	pXNG4 reverse	GTCCATGCCGAGAGTGATCC
