# Supplementary material for: De Novo Synthesis of Phosphatidylcholine Is Essential for the Promastigote But Not Amastigote Stage in Leishmania major
Source: Front Cell Infect Microbiol. 2021 Mar 12;11:647870. doi: 10.3389/fcimb.2021.647870 (PMC7996062; doi:10.3389/fcimb.2021.647870)
Supplement: Supplementary file 7 [file DataSheet_7.pdf]

Table S2. Quantitative analysis of colocalization between GFP-CEPT and the ER marker BiP

GFP-CEPT Image #	# of Cells analyzed	Pearson's Coefficient
1	4	0.809
2	7	0.754
3	5	0.755
4	3	0.68
5	9	0.742
6	3	0.713
7	6	0.756
8	23	0.796
9	15	0.734
10	8	0.698
11	14	0.773
12	10	0.737
13	9	0.783
14	9	0.761
15	6	0.736
16	5	0.519

Total cells analyzed: 136. Average Pearson's Coefficent ± SD: 0.734 ± 0.04
